# From GROMACS Molecular Dynamics Simulations to Electronic Absorption Spectra: A Tutorial for Small Molecules in Organic Solvents

**DOI:** 10.1021/acs.jchemed.6c00154

**Published:** 2026-09-01

**Authors:** Chiara Picarelli, Giuseppina Raffaini, Matteo Tommasini

**Affiliations:** Dipartimento di Chimica, Materiali e Ingegneria Chimica “Giulio Natta”, Politecnico di Milano, 276093 Piazza Leonardo da Vinci, 32−20133 Milano, Italy

**Keywords:** Graduate Education, Laboratory Instruction, Physical Chemistry, Computer-Based Learning, Molecular Mechanics/Dynamics, Quantum Chemistry, Spectroscopy

## Abstract

Classical molecular dynamics is a theoretical method useful for investigating noncovalent intermolecular interactions and conformational flexibility, enabling the simulation of processes such as aggregation/dissolution and adsorption. Molecular dynamics and quantum chemistry are increasingly introduced in chemistry curricula, yet students may encounter difficulties when translating theoretical concepts into robust simulation protocols, particularly outside biomolecular systems. Here, we present a tutorial designed to guide students through the preparation, execution, and analysis of the simulations of small organic molecules in nonaqueous solvents, followed by the calculation of ultraviolet–visible (UV–vis) absorption spectra using time-dependent density functional theory. This tutorial focuses on several practical issues: obtaining force-field parameters, building simulation cells, selecting ensembles, and interpreting critical simulation parameters. Custom solvent models are validated against reference density and radial distribution functions, demonstrating how selected observables can be used to assess simulation reliability. We illustrate the usefulness of molecular dynamics by showing that molecular flexibility and solvent interactions broaden the UV–vis absorption band of indigo in chloroform. This contribution is intended as a hands-on educational resource for upper-level undergraduate and graduate students enrolled in physical chemistry and computational chemistry courses, as well as for Ph.D. students working in chemistry, physics, or material science, who are approaching molecular simulations. To support dissemination, we have released a ready-to-use package on GitHub that includes solvent boxes and all the necessary files and data for incorporating new molecules into GROMACS simulations: https://github.com/cippee/SolventSafari-for-GROMACS.

## Graphical abstract

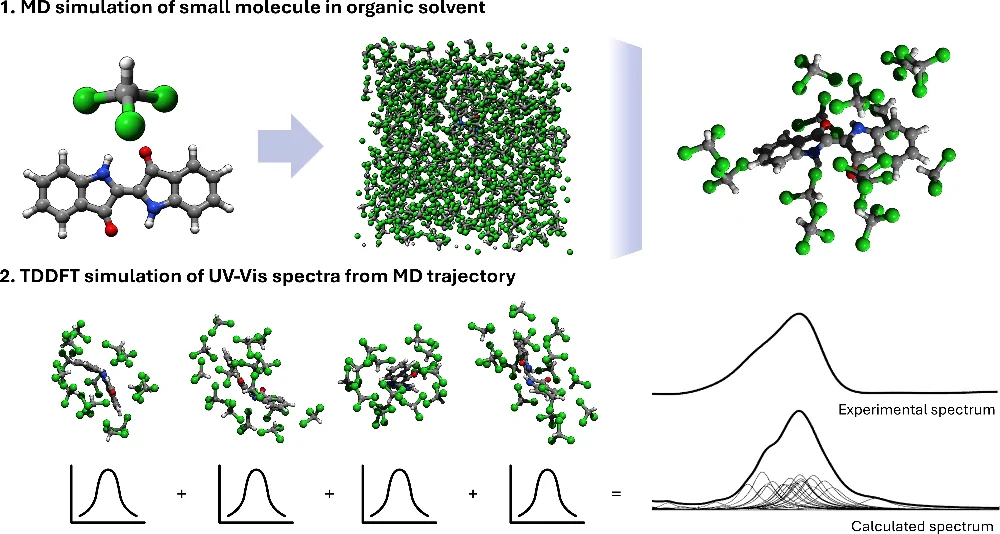

## 1 Introduction

Atomistic simulations are increasingly incorporated into graduate-level courses in physical chemistry, computational chemistry, and materials science, where they support the interpretation of experimental data, e.g., NMR and ultraviolet–visible (UV–vis) spectra.^1–5^ Molecular dynamics (MD) allows students to explore conformational flexibility, solvation, and collective molecular behavior, providing an atomistic description of systems containing a large number of molecules, making it a versatile tool for studying small molecules, macromolecules, and materials.

MD simulations provide important geometric descriptors, including radial distribution functions (RDFs or *g*(*r*)), which characterize solvation shells^6–10^ and intermolecular organization. Analyses of MD data can help rationalize macroscopic properties, such as density, and dynamic phenomena, such as diffusion.^11–15^ MD is, therefore, the method of choice for simulating the collective behavior and time-dependent properties of molecular systems. In this context, it is useful to distinguish between the description of the potential energy and the method used to propagate a molecular system in time. Here, the potential energy surface is described within a classical framework (commonly referred to as molecular mechanics (MM)), where the energy is expressed through force fields, atoms are treated as beads connected by springs that model the covalent bonds, and the electronic degrees of freedom are not explicitly included. Molecular dynamics refers to the numerical integration of equations of motion (typically Newton equations), which allows the system to evolve on a given potential energy surface. Hereafter, MD refers to classical atomistic molecular dynamics based on force fields.

MD simulations are often introduced in the context of biomolecular systems, for which established force fields, tutorials, and workflows are readily available. Extensive efforts have been devoted to the development and refinement of force fields for different classes of biomolecules, including proteins, lipids, and carbohydrates.^16–18^ Among the available MD engines, GROMACS stands out for its efficiency^19,20^ and is widely adopted for biomolecular simulations in water with well-documented workflows.^21^ However, students working in physical chemistry and spectroscopy are often faced with systems that fall outside the biomolecular paradigm (i.e., non-amino-acid-based molecules dissolved in nonaqueous organic solvents, a scenario commonly encountered in materials science and physical chemistry). For these systems, practical and pedagogical guidance is scarce. Although resources provide parameter files for several common solvents and molecules,^22^ they rarely offer a general, transferable protocol for building new ones. Thus, preparing the simulation inputs may become arduous, discouraging beginners.

This tutorial is intended for, but not limited to, graduate and early-stage doctoral students learning to perform molecular simulations in a research-oriented context and to experimental spectroscopists interested in understanding simulation protocols underlying spectroscopic predictions. The workflow integrates MD simulations with time-dependent density functional theory (TD-DFT) calculations to model UV–vis absorption spectra in solution, using Q-Force^23^ to derive force field parameters, GROMACS^20,24^ for MD simulations, Gaussian^25^ for TD-DFT calculations, and VMD^26^ for trajectory visualization and analysis. The explanatory case study of indigo dissolved in chloroform guides students from input files and parameters to practical computer commands, thus linking theory to the computational workflow.

In Section 2, we present the conceptual framework underlying molecular simulations, including the rationale behind common methodological choices. Readers primarily interested in practical instructions can proceed directly to Section 3, which focuses on building a molecular model, topology generation, force-field assignment, and setting up the simulation cell. In Section 4 we apply the workflow to compute the UV–vis absorption spectrum of indigo in chloroform, and in Section 5, we provide concluding remarks.

Upon completion of this tutorial, students should be able to (i) build and parametrize small organic molecules; (ii) set up and run MD simulations in GROMACS; (iii) validate the models against experimental observables such as density and radial distribution functions; and (iv) compute UV–vis spectra using TD-DFT of MD-derived microstates. More broadly, the tutorial aims to develop the ability to critically assess simulation parameters and understand how methodological choices affect the physical meaning of the results.

## 2 The Framework of Molecular Simulations

^27^ Every simulation requires assumptions about the system being modeled that are encoded in specific input parameters. In GROMACS, the molecular dynamics parameters are collected in the *mdp* files (see ). This section describes the main parameters to be defined in the *mdp* files and their physical rationale.

### 2.1 Energy Minimization

Energy minimization is used to find a stable geometry that maximizes favorable intermolecular interactions and reduces strains. In practice, unfavorable interactions, arising from steric hindrance, often occur during the construction of the simulation cell, where molecules are usually positioned according to geometric or stochastic criteria rather than energetic considerations. Random insertion of solvent molecules, as well as the use of hypothesized molecular structures/conformations, can therefore result in atomic overlaps or highly strained intramolecular configurations.

Energy minimization moves the atoms of the system toward a local minimum of the global potential energy surface, preserving the overall molecular structure. In GROMACS, convergence is controlled by the force tolerance (“emtol”) and maximum atomic displacement per iteration (“emstep”). Minimization can be performed using the steepest descent method, which is a robust method for removing large unfavorable contacts, or the conjugate gradient method, which converges more efficiently near a minimum. If the energy minimization is performed before running the proper MD simulation, moderately stringent parameters (“emtol” ca. 0.05–0.1 kJ mol^–1^ nm^–1^, “emstep” ca. 0.01–0.05 nm) should be sufficient; stricter criteria may be applied for retrieving well-relaxed structures. This is typically performed on the system after the MD run. Minimization should be interpreted not as a means of finding a global minimum, but as a practical step to ensure physically reasonable starting configurations for MD simulations.

### 2.2 Integrator of the Equations of Motion

In MD simulations, atomic positions and velocities are propagated over time by numerically integrating the classical Newton’s equations of motion.

The velocity Verlet^28^ algorithm is a commonly used deterministic integrator in classical MD. In velocity Verlet, positions and velocities are updated using forces evaluated at well-defined timesteps. Stochastic integrators (e.g., Langevin^29^), add friction and random forces that represent the coupling to an implicit heat bath, naturally sampling the canonical ensemble (constant number of particles, volume and temperature, see below) and often improving equilibration in systems with slow dynamics. The fundamental distinction between deterministic and stochastic integrators lies in the presence of random forces. Velocity Verlet preserves time reversibility and phase-space structure; Langevin dynamics introduces controlled noise and dissipation.

### 2.3 Integration Time Step

The integration time step directly affects numerical stability and physical accuracy of an MD simulation.^30^ A time step of 1 fs (10^–15^ s) allows all bond vibrations to be explicitly resolved, including those involving hydrogen atoms (freq = *v* = 10^14^ s^–1^) and ensures stable energy conservation without constraints.^31^ A 2 fs time step halves the computational cost but requires constraints on high-frequency motions to avoid instabilities; accordingly, larger timesteps are typically combined with constraints. A 1 fs time step represents a common compromise for unconstrained simulations. Timesteps smaller than 1 fs can be used to better resolve fast vibrational motions and improve energy conservation, but at a higher computational cost.^30^

### 2.4 Thermodynamic Ensemble and Temperature Control

In MD simulations, a thermodynamic ensemble defines which macroscopic variables are kept constant and specifies the conditions under which the system is studied. The choice of the ensemble depends on the purpose of the simulation and generally follows a well-defined sequence during system preparation and production run. In the canonical ensemble (NVT), the number of particles (*N*) and the volume (*V*) remain fixed, while a thermostat keeps the system at the target temperature (*T*), constant on average, by exchanging kinetic energy with an external heat bath. NVT is used for rapid thermalization while preserving the initial box geometry, i.e., to stabilize the system temperature without altering the volume or density. The isothermal–isobaric (NPT) ensemble conserves the number of particles, pressure (*P*), and temperature, combining a thermostat and a barostat. The NPT ensemble allows the simulation box to change size, enabling density equilibration at a given temperature and pressure, and it is particularly indicated for condensed-phase systems. The microcanonical (NVE) ensemble conserves the total energy (*E*) of the system and does not employ thermostats or barostats. Consequently, temperature and pressure fluctuate naturally. NVE simulations are primarily used to analyze intrinsic dynamical properties or to assess the numerical quality of the integration scheme. Since in NVE, no external control is applied, the numerical stability hinges on the time step. Excessive drift in temperature or pressure indicates the accumulation of integration errors and the necessity of re-equilibration.

A typical simulation workflow may therefore consist of four stages: (1) energy minimization, (2) NVT equilibration to reach the target temperature *T*, (3) NPT equilibration to reach the correct density, and (4) the production run in the appropriate ensemble for the properties of interest. The equilibration time (stages 2 and 3) depends on the system conditions and should be verified by monitoring an adequate physical quantity, e.g., total energy or kinetic energy. For the systems considered here, under standard conditions, equilibration typically occurs within 200 ps. This workflow is implemented in the example input files provided in .

### 2.5 Nonbonded Interactions and Cutoff Schemes

Nonbonded interactions, i.e., van der Waals and electrostatic forces, dominate intermolecular organization and must be treated carefully to ensure both numerical stability and physical accuracy. In MD simulations, these interactions are evaluated explicitly within a finite cutoff distance, which defines the spatial range over which particles interact. In other words, if the distance between two atoms is larger than the cutoff distance, their interaction is neglected in the calculation. The cutoff choice, therefore, represents a balance between computational efficiency and the faithful representation of intermolecular forces.

van der Waals interactions decay rapidly, proportionally to 1/*R* ^6^, and can therefore be truncated at 1.0–1.2 nm. To prevent discontinuities in the forces, switching or shifting functions smooth the potential near the cutoff.^32^ Electrostatic interactions decay much more slowly, proportionally to 1/*R*, and cannot be reliably truncated at short distances. For this reason, electrostatics are treated using the Particle Mesh Ewald (PME) method^33^ computed with the Particle–Particle Particle-Mesh (P3M) scheme.^34^ PME splits the Coulomb interaction into a short-range real-space contribution and a long-range reciprocal-space term evaluated on a grid. With PME, moderate real-space cutoffs (ca. 1.0–1.2 nm) are sufficient; further increasing them does not improve accuracy, but may significantly increase computational cost. When using periodic boundary conditions (see below), the dimension of the cell should be at least twice the cutoff distance to avoid artifacts caused by self-interaction with the mirror images. PME also guarantees that long-range electrostatic interactions are treated consistently under periodic boundary conditions, preventing unphysical artifacts that would arise from simple truncation.

Cutoff distances define the maximum range at which intermolecular forces are explicitly calculated, while neighbor lists, which track all atomic pairs (*i* < *j*) within the cutoff (*r*_*ij*_ < *r*_cutoff_), ensure that these interactions are evaluated efficiently during the simulation. Appropriate cutoff and electrostatic methods enable stable simulations that capture the intermolecular interactions.

### 2.6 Periodic Boundary Conditions

Focusing on liquids, the periodic boundary conditions (PBC) model bulk systems by replicating the simulation box in space, eliminating surface effects, and enabling the calculation of bulk structural and thermodynamic properties. Since particles should not interact with their nearest images, the simulation box must be large enough to prevent spurious self-interactions, especially for long-range nonbonded forces (Figure 1). Practically, provided that the box edges satisfy the cutoff distance, a cubic box with edge lengths equal to the molecular size plus approximately 1 nm is often sufficient, i.e., *L*_box_ > 2*r*_cutoff_ and *L*_box_ > *l*_mol_ + 2 nm.

**Figure 1:**
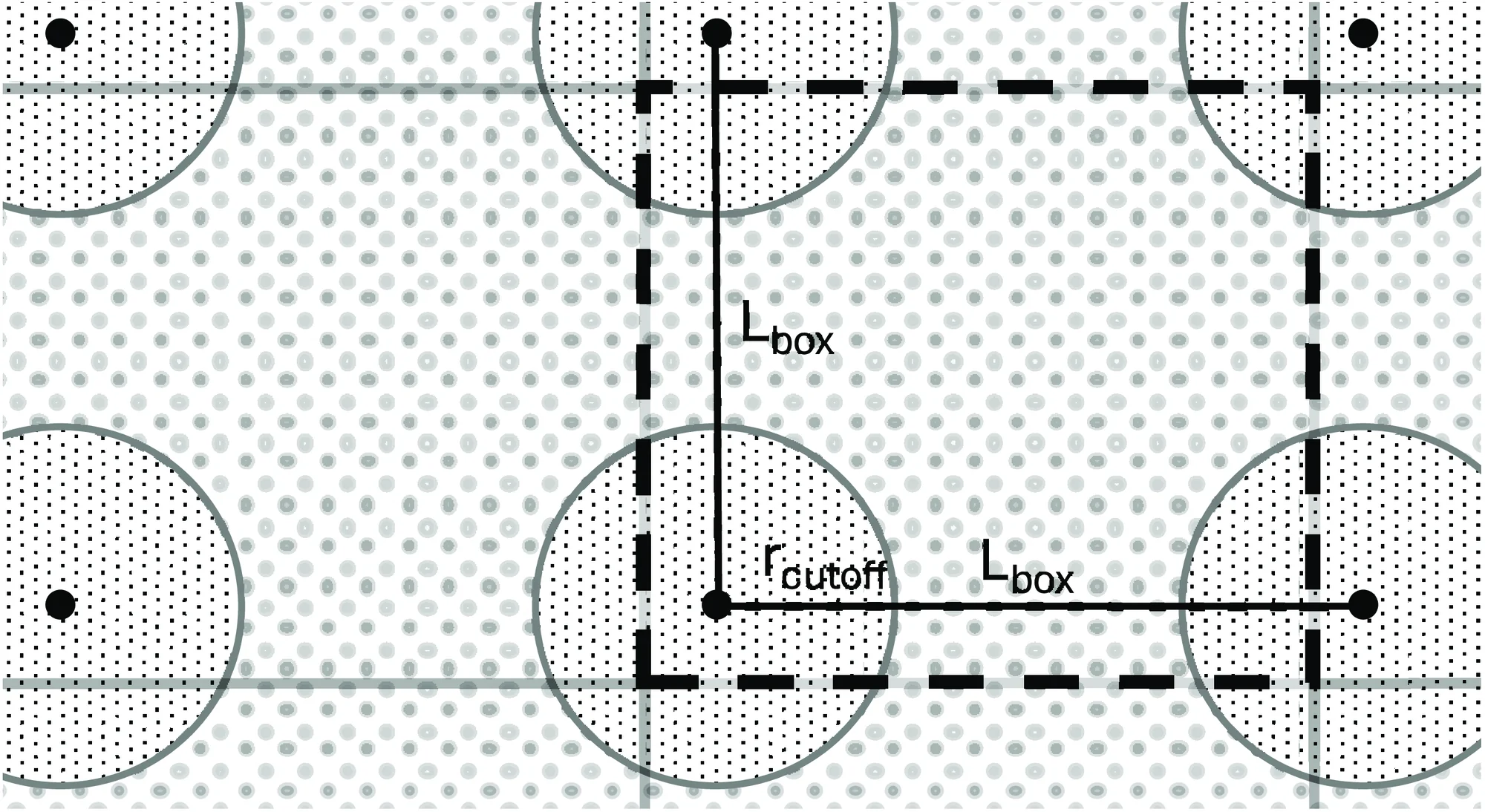
Schematic representation of a simulation cell under PBCs, with a distance between replicas, which is equal to the box edge larger than twice the cutoff distance (*L*_box_ > 2*r*_cutoff_).

Having introduced the main parameters governing a molecular simulation (e.g., integrators, time step, thermodynamic ensemble, nonbonded interactions, and periodic boundary conditions), students should critically assess which approximations are appropriate for their specific system: should it exchange energy with a thermal bath, or evolve as isolated? Should the volume fluctuate? How appropriate are the cutoffs and time step for the phenomena being simulated?^27^ Reflecting on these questions, helps ensure that simulations are not only numerically stable but also physically meaningful, and cultivates a deeper understanding of the assumptions underlying the computational model. In practice, all these choices are implemented concretely in the simulation input (i.e., GROMACS *mdp* files; see ), where physical approximations are translated into computational parameters.

## 3 Setting Up the Simulation

Having established the framework for MD simulations, this section focuses on the definition of atom types, bonded and nonbonded parameters, and the construction of the topology file, which together specify the molecular model. Accurate coordinates, topology, and force-field parameters must be chosen carefully to ensure a stable trajectory that reflects the intended system.

To run an MD simulation, three key components are required: the initial atomic coordinates, the potential, commonly referred to as the force field, and the molecular connectivity encoded in the topology file. The initial coordinates can be acquired from experimental techniques,^35,36^ or generated using molecular modeling tools (e.g., Avogadro^37^). The potential, or force field, describes the intermolecular and intramolecular interactions that dictate the forces acting on each atom through bonded and nonbonded interactions. The force field is implemented through a set of parameters that describe bond stretching, angle bending, torsional rotations, and nonbonded interactions such as van der Waals and electrostatic forces.^38^ Hence, the total potential energy *V*_tot_ of the system is commonly expressed as a sum of such contributions:^39^

1 $$V_{\mathrm{tot}}=\underset{\mathrm{r bonds}}{\sum }K_{r}{\left(r-r_{\mathrm{eq}}\right)}^{2}+\underset{\mathrm{\Theta angles}}{\sum }K_{\theta }{\left(\theta -\theta_{\mathrm{eq}}\right)}^{2}+\underset{\mathrm{\phi dihedrals}}{\sum }\frac{V_{n}}{2}\left[1+\cos\left(n\phi -\gamma \right)\right]+\underset{i<j}{\sum }\left[\frac{A_{ij}}{R_{ij}^{12}}+\frac{B_{ij}}{R_{ij}^{6}}+\frac{q_{i}q_{j}}{\epsilon R_{ij}}\right]$$

where the first three terms account for bonded interactions (i.e., bond stretching, angle bending, and torsional rotations around dihedral angles), whereas the last term represents nonbonded interactions, typically modeled through a Lennard-Jones potential for van der Waals forces and a Coulombic term for electrostatics. Although functional forms and parameters vary across different force fields (e.g., AMBER, CHARMM, OPLS), this expression captures the essential physical contributions governing molecular mechanics simulations.

In GROMACS, the molecular connectivity and all force field parameters associated with a given system are collected in the so-called topology file.^40^

### 3.1 The Topology and the Force Field Parameters

The topology of a molecule describes its connectivity, i.e., how atoms are linked by bonds, how they form angles and dihedrals, and it provides the parameters that govern these interactions. The topology is the blueprint that connects the chemical structure to the mathematical model used in the simulation. In GROMACS, the topological information is organized using two related files: the topology file (*top*) and the included topology file (*itp*). The *top* file defines the complete molecular system to be simulated and may include one or more *itp* files, which contain the detailed definitions of individual molecular species or force-field components. Together, these files specify the molecular composition of the system, the force field parameters, and how different molecular entities are combined within the simulation box.

A central concept in classical MD simulations is the *atom type*. Importantly, the atom type does not correspond uniquely to a chemical element, but rather to a specific chemical environment defined by the force field. For example, carbon atoms in aromatic rings, aliphatic chains, or carbonyl groups are treated as distinct atom types, even though they are all chemically carbon. Each atom type is associated with a specific set of bonded and nonbonded parameters (e.g., Lennard-Jones σ and ε values), linking the chemical structure of the molecule and the mathematical form of the force field. Correct atom type assignment is therefore essential to obtain physically sound results.

GROMACS supports several widely used force fields, including CHARMM, AMBER, and OPLS, each with a library of predefined atom types and interaction parameters. For instance, in proteins atom typing and topology generation are often automated through residue definitions encoded in these libraries.^36,41^

Regardless of whether parameters are defined explicitly or retrieved from a force-field library, GROMACS topology files are structured into a set of well-defined sections, each controlling a specific aspect of the molecular model. These sections are briefly commented in Table 1.

**Table 1:** Scheme with the Sections of the GROMACS Topology (top) and Included Topology (itp) Files

Section	Content
[defaults]	Specifies global settings for nonbonded interactions, including the functional form of the potential, combination rules for Lennard-Jones parameters, and scaling factors for 1–4 interactions.
[atomtypes]	Specifies the atom type labels and assigns the nonbonded interaction parameters σ and ε for the Lennard-Jones potential. Charges and masses are initialized here but assigned explicitly in the [atoms] section.
[nonbond_params]	Allows explicit definition of nonbonded interaction parameters for specific pairs of atom types, overriding default combination rules (e.g., geometric averages of σ_*ii*_, σ_*jj*_, ε_*ii*_, and ε_*jj*_ in OPLS-aa).
[moleculetype]	Specifies the name of the molecule and the exclusion rules for nonbonded atoms (e.g., “nrexcl = 3” excludes nonbonded interactions between atoms separated by less than three bonds). For instance, this option implements the exclusion of unphysical van der Waals interactions among bonded atoms.
[atoms]	Assigns atom indices, types, charges, and masses to each atom in the molecule.
[bonds] and [angles]	Define bonded interactions using harmonic potentials.
[dihedrals]	Specifies potential using Fourier coefficients to describe rotational barriers.^42,43^
[system]	Provides a descriptive name for the simulated system.
[molecules]	Specifies the molecular composition of the simulation box by listing each molecular species and the corresponding number of molecules.

When constructing topologies for small organic molecules, two conceptually distinct approaches can be adopted, both illustrated in Figure 2 using chloroform as an example. In the first approach (panel (a) in Figure 2), the topology file contains a fully custom definition of the force field. Sections such as [defaults], [atomtypes], and [nonbond_params] are explicitly included, and all bonded and nonbonded parameters are defined directly within the topology file. This yields a transparent, self-contained representation of the molecular model, in which all parameters are visible. In the second approach (panel (b) in Figure 2), the topology file is defined using a native GROMACS force field. The top file includes the force-field itp library file (e.g., oplsaa.ff/force fielditp), and the sections [atomtypes] and [nonbond_params] are omitted, since these are read directly from the library files. In both cases, any parameter explicitly defined in the topology file overrides the corresponding value in the force-field library. Overriding is useful for fine-tuning but should be applied with caution to preserve the internal consistency of the force field.

**Figure 2:**
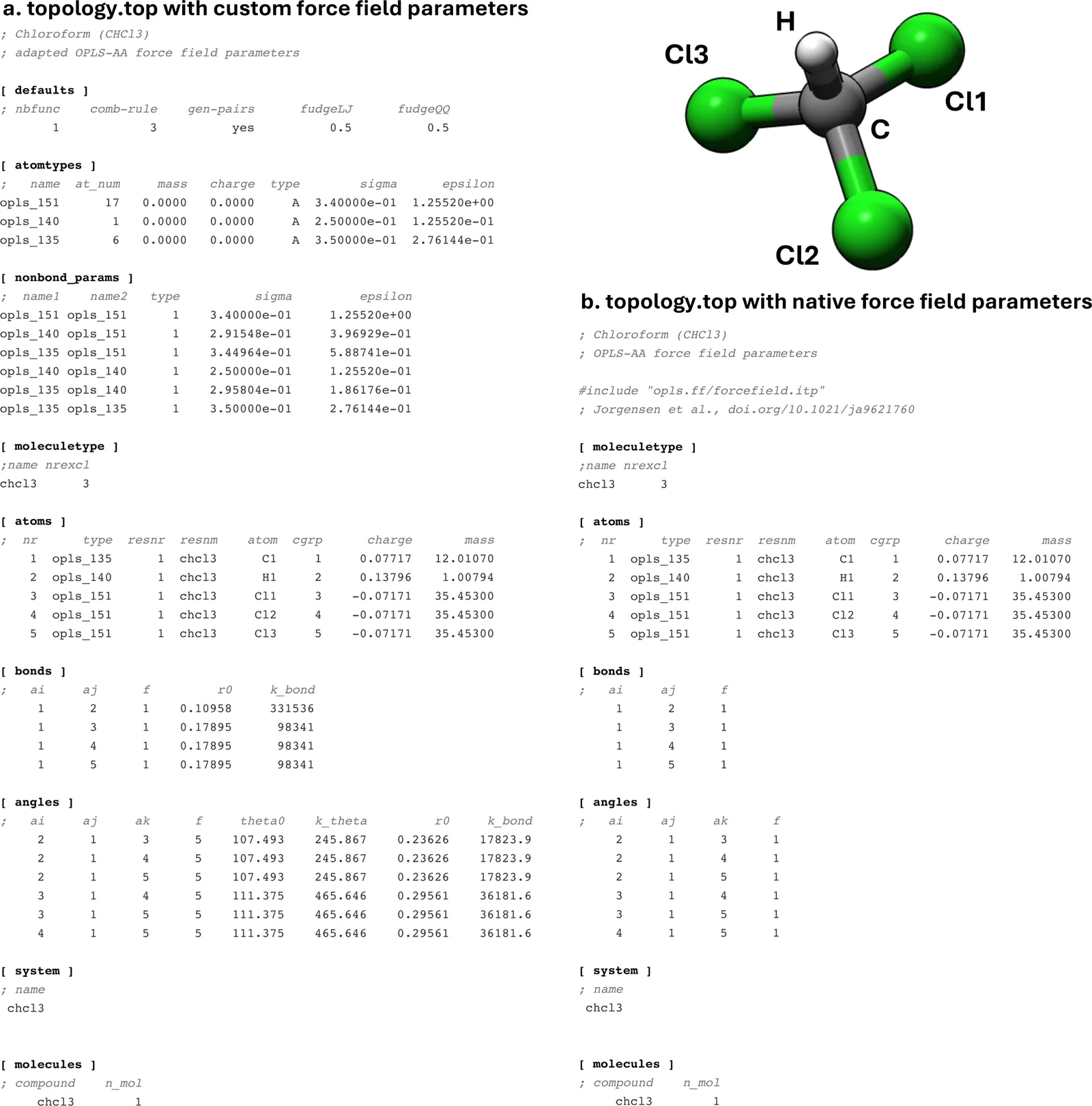
Definition of the molecular structure and topology of chloroform as required by GROMACS. In the fully custom implementation (panel (a)), atom types, van der Waals parameters (σ and ε), partial charges, and bonded parameters (bond lengths, angles, and dihedrals) are explicitly defined. In the library-based implementation (panel (b)), atom types and nonbonded parameters are inherited from the OPLS-aa force field, while bonded interactions are assigned automatically from the corresponding force-field definitions. Unless otherwise specified, partial charges are derived from DFT calculations (scaled Hirshfeld electrostatic charges^44^).

### 3.2 Parametrizing Small Molecules

When simulating molecules that do not contain predefined moieties with associated force field parameters,^35,36^ the user must explicitly define atom types, bonded interactions, and partial charges. Manual topology building is possible, but often error-prone and time-consuming. Automatic tools such as CGenFF (for the CHARMM force field) can assist with topology generation,^40^ but depend on the available atom. To overcome this limitation, here we use Q-Force^44^ with default settings to derive OPLS-aa (i.e., Optimized Potentials for Liquid Simulations-all atom) force field^43,45^ parameters by running quantum mechanical (QM) DFT calculations (PBEPBE/6–31+G­(d) level of theory, Gaussian^25^). In Q-Force, the PBE functional is employed as a default to derive force field parameters, providing a reasonable balance between computational cost and accuracy for structural and vibrational properties (e.g., the frequencies computed with the PBE functional are very close to experimental values^46^). While PBE is known to have limitations in describing dispersion interactions and reaction energetics,^47^ it is generally adequate for generating force field parameters for nonreactive, ground-state molecular systems.

Starting from a molecular geometry (i.e., an *xyz* file), Q-Force produces three essential components required to run a GROMACS simulation: a coordinate file (*gro*), the system topology file (*top*), and the included topology file (*itp*). This is straightforward and documented on the Web site.^48^ From the command line, one first executes “qforce molecule.xyz -o optionfile”. This generates the input files for a QM geometry optimization and the Hessian calculation (e.g., a combined “opt freq” job in Gaussian jargon). This allows Q-Force to determine the bond orders and the force constants. At this point, Q-Force is run again (“qforce molecule -o optionfile”) to generate molecular fragments for parametrizing dihedral angles.^44^ Q-Force then analyzes all QM results to produce the complete topology files (*itp* and *top*), including the force field parameters, and a coherent structure file (*gro*) ready for GROMACS simulations.

Note that Q-Force stores the connectivity and force field parameters within the *itp* file, which is included in the *top* file, with two main advantages:

(i) The *top* file remains compact and organized, useful for large systems with multiple molecules.
(ii) Each *itp* file is self-contained and reusable across different simulations.

Figure 3 illustrates this concept using the *itp* (left) and *top* (right) files produced with Q-Force for the ethanol molecule.

**Figure 3:**
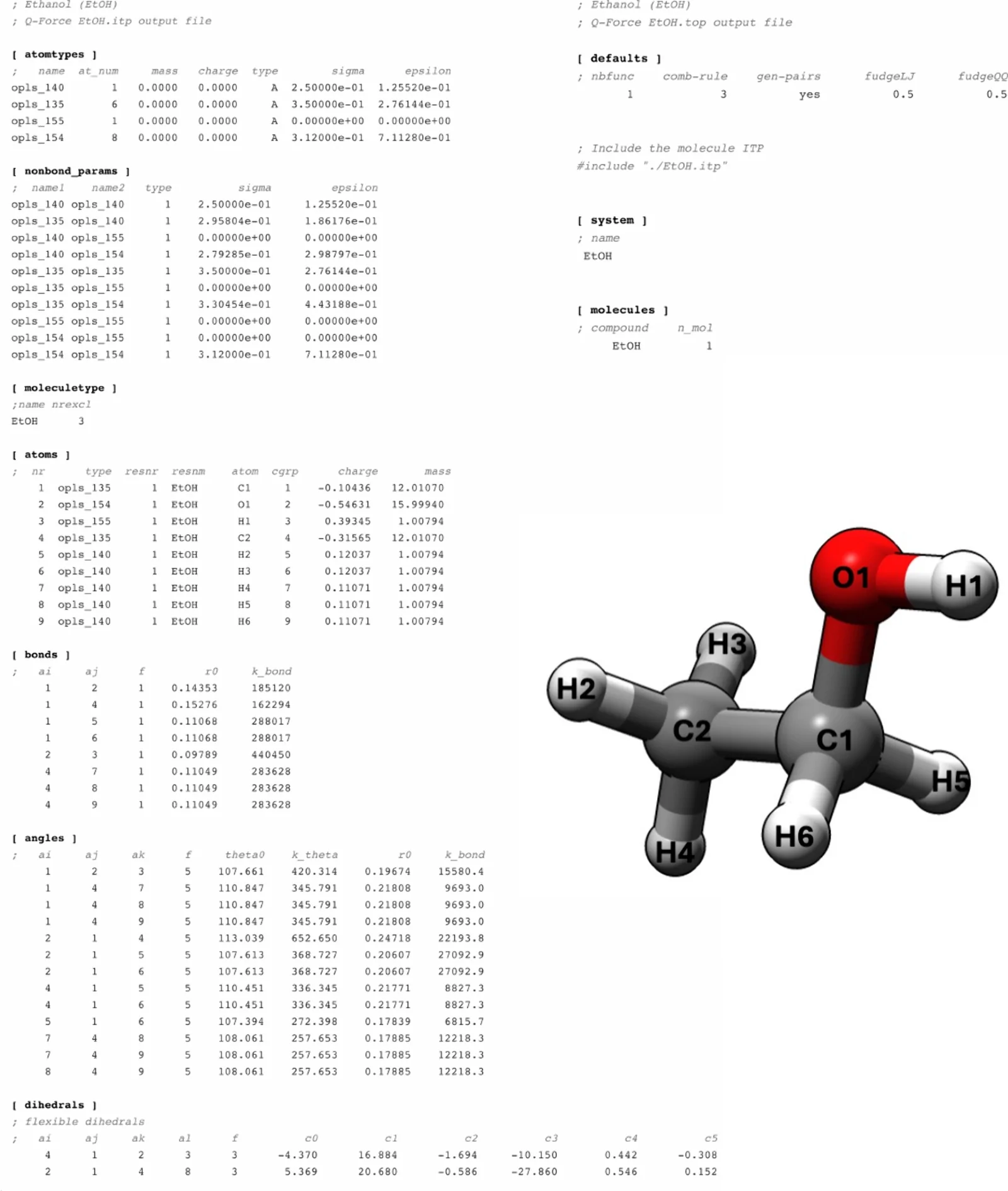
Snapshots of the EtOHitp file (left) and EtOHtop file (right) as produced by Q-Force for the ethanol molecule. Ethanol was selected as an illustrative example, because, unlike chloroform, it includes dihedral terms, allowing a more complete representation of the topology file structure.

Q-Force derives bonded parameters and partial charges from quantum mechanical calculations on molecular fragments, enabling parameter transferability across similar moieties in different molecular systems.^44^ In practice, not only can the *itp* file of a given molecule be reused across different simulations but parameters are also automatically assigned when Q-Force identifies molecular fragments analogous to previously resolved cases. When multiple molecular species are present, the [atomtypes] and [nonbond_params] sections must be defined (or included) at the beginning of the *top* file, moving all atom types and nonbonded parameters from the constituent *itp* files. This is because GROMACS requires that these definitions appear sequentially at the beginning of the *top* file to correctly read and apply the force field across the entire system. See related files in .

Classical force fields, including OPLS-aa, rely on fixed functional forms (such as eq 1) that do not account for electronic polarization or charge transfer, limiting applicability to systems where these effects are irrelevant. To accurately simulate such effects, one should use polarizable force fields.^49,44^

Regardless of how force field parameters are generated and assigned, basic integrity checks should be carried out to verify their physical soundness before starting any simulations. A simple and instructive validation strategy is to analyze vibrational peak positions from a short, tightly sampled MD (e.g., 0.05–0.1 fs over a few ps) and examine vibrational peak positions. From such a trajectory, an approximated vibrational spectrum can be obtained using tools such as VMD (Analysis > IR Spectral Density Calculator). Note that this MD-derived spectrum is not an infrared spectrum in the strict sense since intensities do not come from dipole-moment derivatives, but peak positions are a robust qualitative check of bond/angle force constants. To perform this analysis, both the structure (*gro*) and trajectory (*trr*) files are loaded in VMD, and atomic charges must be assigned via the command line in the VMD terminal:

> set sel [atomselect top ″all″]

> $sel set charge {charge_atom1 charge_atom2···}

In Figure 4, three spectra are shown for comparison: the DFT-calculated IR spectrum of ethanol (bottom in green), available from the Q-Force Hessian calculation, and the VMD-calculated spectra from the MD trajectories with a customized OPLS-aa force field (middle in red) and native OPLS-aa parameters (top in blue). This comparison serves as a qualitative check to verify that the force-field parameters produce reasonable vibrational frequencies, which confirms that bond and angle force constants are correctly assigned.

**Figure 4:**
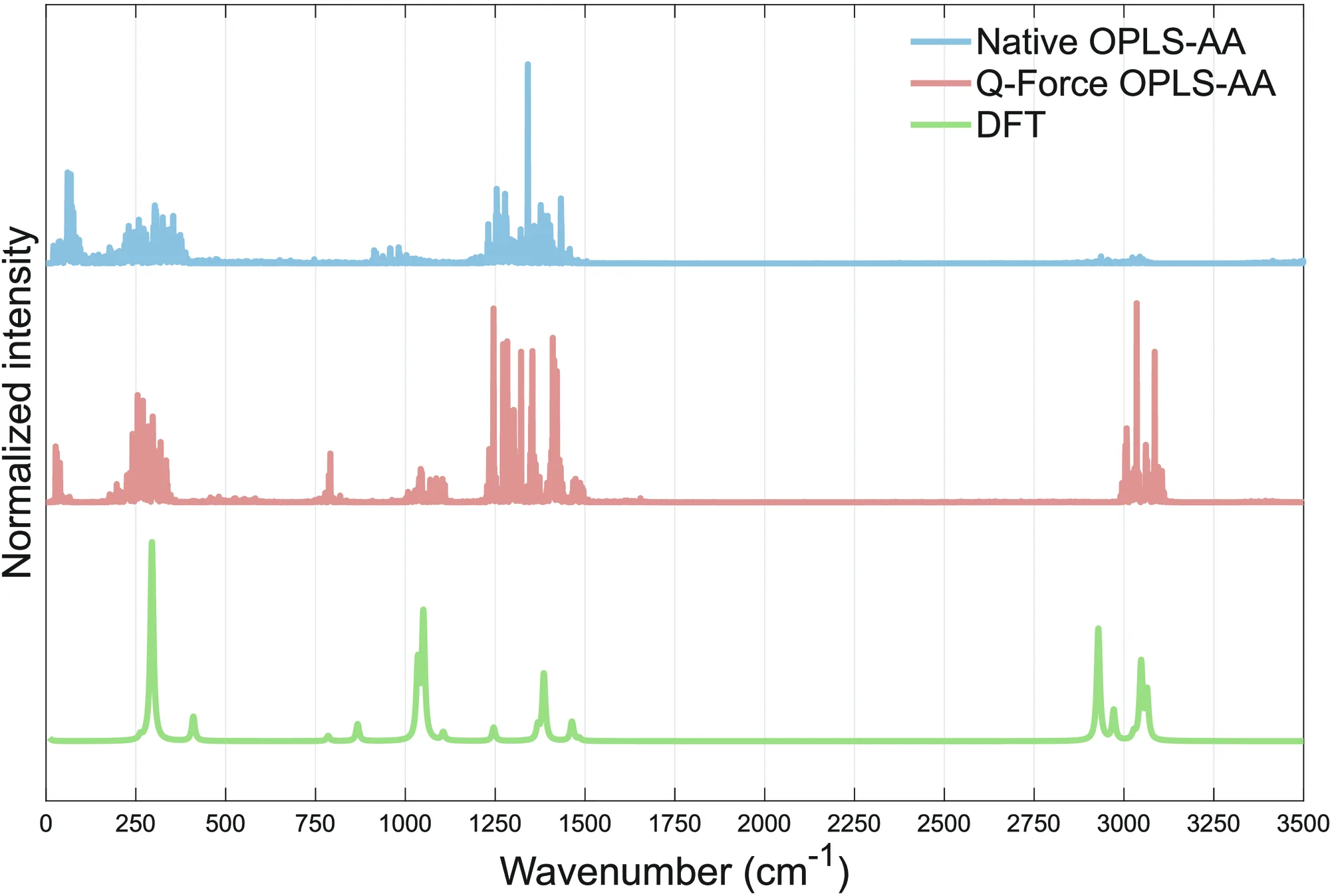
IR spectral densities of states of ethanol: the blue curve represents the IR spectral density of ethanol simulated in GROMACS using the native OPLS-AA force field. The red curve corresponds to simulations performed with modified OPLS-AA parameters obtained via Q-Force. The green curve, shown at the bottom, reports the IR spectrum of ethanol calculated with Gaussian at the PBEPBE/6–31+G­(d) level of theory. The red curve shows improved agreement with the quantum-mechanical spectrum (green curve) compared to the standard OPLS-AA model.

Constructing a realistic solvent model requires reference data to ensure and validate its correct behavior. In GROMACS, the experimental isothermal compressibility (β) is a required input parameter for the barostat, as it determines the magnitude of volume fluctuations the system can tolerate. The experimental density (ρ) and the radial distribution function (*g*(*r*)) serve as benchmarks to assess, a posteriori, the reliability of the simulation. Ethanol (Figure 5, top panels) and chloroform (Figure 5, bottom panels) were selected to allow comparison with the published radial distribution functions.^50,51^

**Figure 5:**
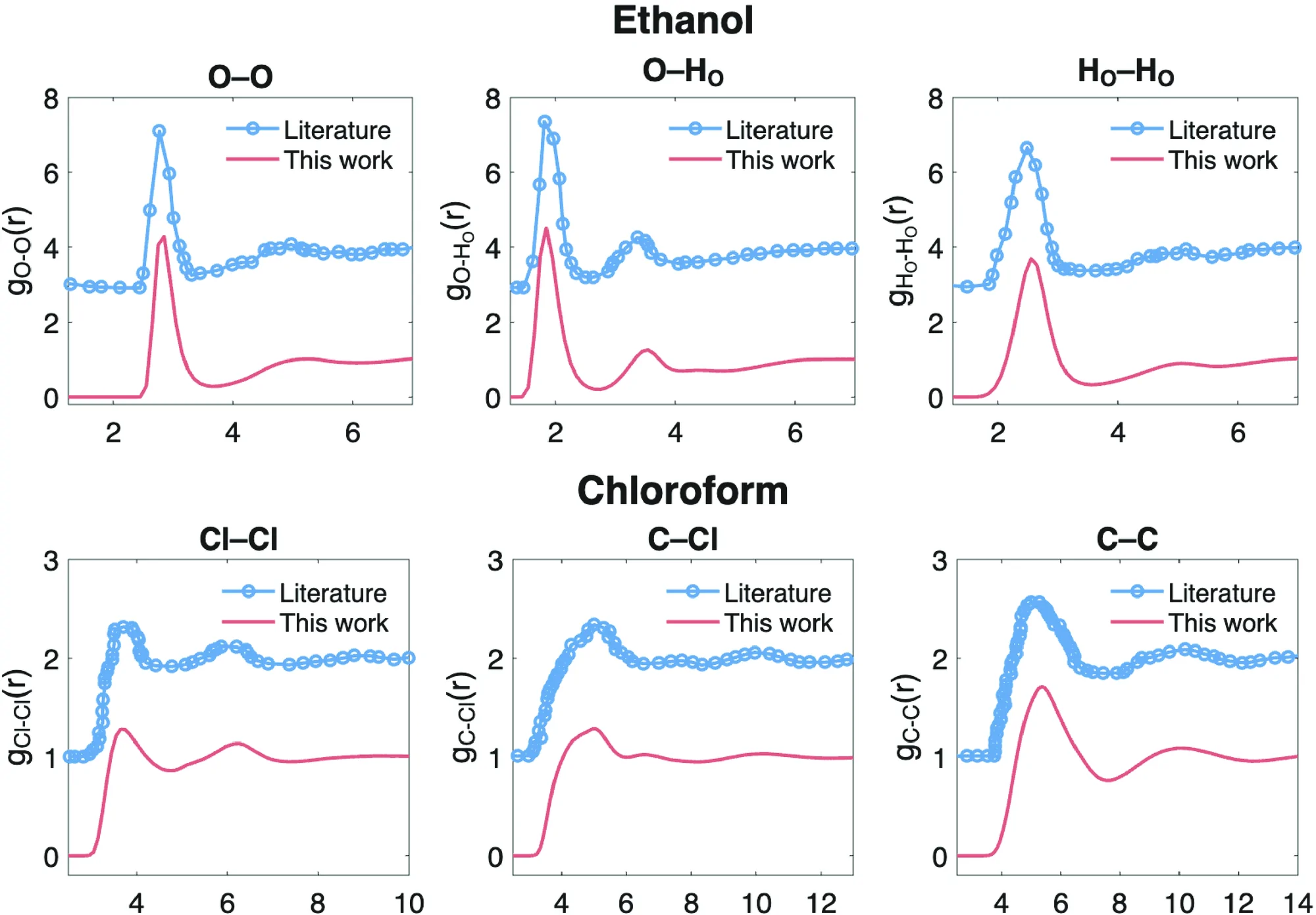
Partial radial distribution functions g­(r): results from this work (red lines) compared with literature data^50,51^ (blue lines. Top panels: ethanol, literature data digitized from Figure 5b of ref 50. Bottom panels: chloroform, literature data digitized from Figure 6 of ref 51

To proceed with the tutorial, we selected some of the most used solvents in spectroscopy (see Table 2), adopting the experimental density and isothermal compressibility data published by Marcus in a popular reference book.^52^ The complete input files, simulation box details, and simulation outputs are provided in and on GitHub, allowing readily adoption or adaptation of the protocol. These input files can also serve as a template for classroom exercises. Instructors and students may select from them for practical training and assignments.

**Table 2:** Complete List of the Solvents We Simulated, with the Corresponding Labels and Experimental Data of Density and Isothermal Compressibility^a^

	Experimental density (kg/m^3^)	Isothermal compressibility (× 10^–4^bar^–1^)
Acetone, Ac	785	1.324
Acetonitrile, ACN	776	1.070
Tetrachloroethylene, C_2_Cl_4_	1615	0.791
Tetrachloromethane, CCl_4_	1584	1.080
Chloroform, CHCl_3_	1479	1.033
Cyclohexane, cHex	774	1.140
Chlorobenzene, ClPh	1101	0.771
Carbon disulfide, CS_2_	1253	0.950
Dichloromethane, DCM	1394	1.026
Dimethyl sulfoxide, DMSO	1096	0.524
Ethanol, EtOH	785	1.153
Methanol, MeOH	787	1.248
Hexane, nHex	655	1.706
Benzene, Ph	869	0.962
Tetrahydrofuran, THF	884	0.953
Toluene, Tol	862	0.922

a As reported by Marcus.^52^ For each solvent listed in this table, the complete input and output files are available in the .

### 3.3 The Simulation Cell

For each solvent, we built a cubic box containing 9000 atoms, with GROMACS modules “gmx editconf” and “gmx insert-molecules”. This choice was dictated by the fixed-format structure of the .gro file, which imposes an upper limit of 9999 atoms. The number of molecules, therefore, varies; e.g., chloroform and ethanol boxes contain 1800 and 1000 molecules, respectively, reflecting the different molecular sizes.

We determined the size of the box edge *l*_box_ from the experimental density ρ using the equation below:

$$\rho =\frac{m}{V_{\mathrm{box}}}=\frac{n\left(M_{w}/N_{\mathrm{Av}}\right)}{l_{\mathrm{box}}^{3}}\to l_{\mathrm{box}}=\sqrt[{3}]{\frac{M_{w}}{N_{\mathrm{Av}}}\times n\times \frac{1}{\rho }}$$

given the mass *m*, the chosen number of molecules *n* in the box, the molecular weight of the molecule *M*_*w*_, and Avogadro’s number *N*_Av_.

After conjugate-gradient minimization, the system was equilibrated for 1 ns: 500 ps in NVT using the velocity-rescaling thermostat,^53^ followed by 500 ps in NPT at 1 bar using the same thermostat and the cell-rescaling barostat.^54^ The subsequent NPT production simulation spanned 5 ns.

includes the “ethanol_solvent” directory with the workflow script (Figure 6) and the associated parameter files (*mdp*).

**Figure 6:**
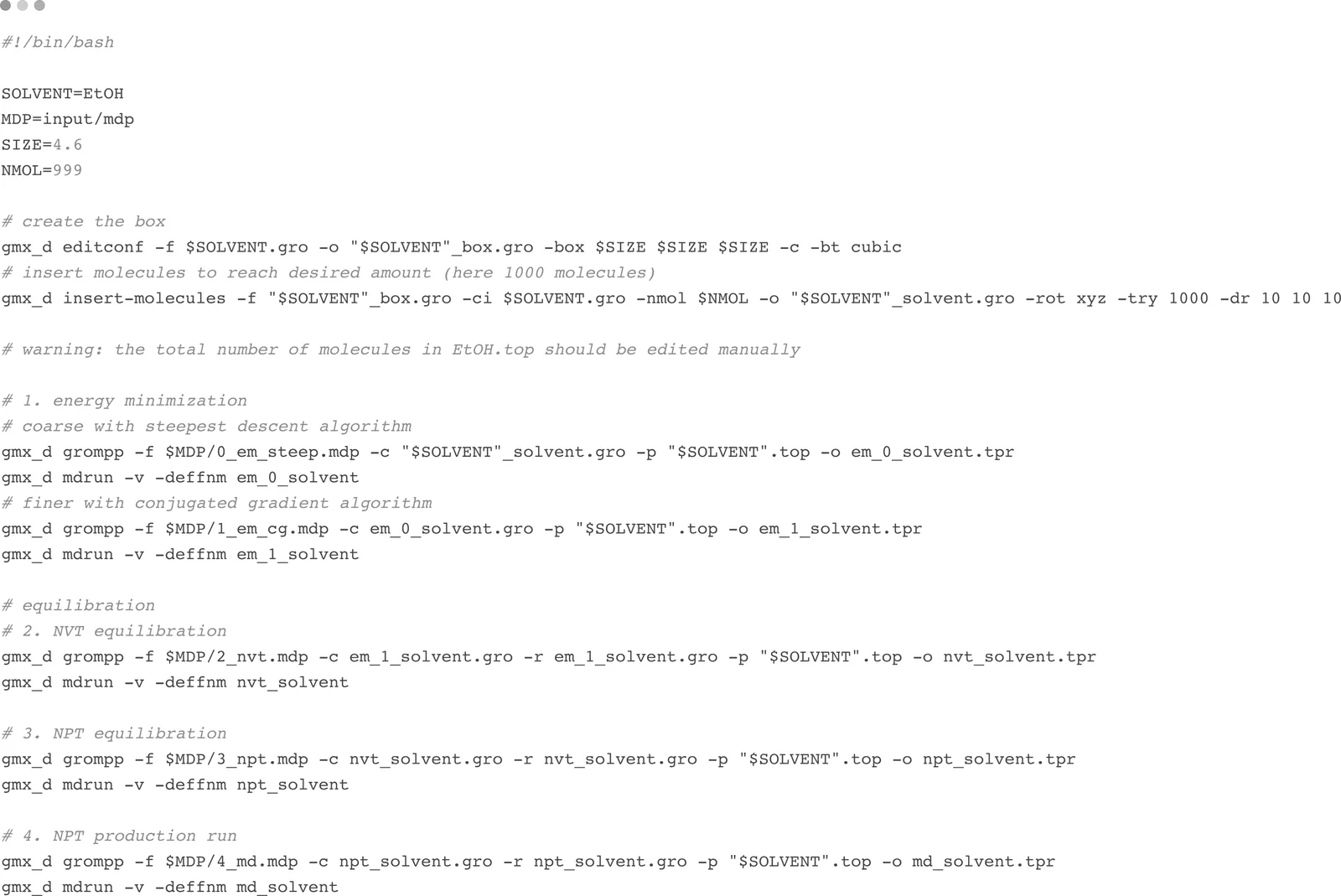
Script with typical workflow and parameters for simulating ethanol. Note that all GROMACS steps are run calling “gmx_d”, where “d” stands for the double precision version of the software.

This protocol was applied to all solvents listed in Table 2; simulation results are reported in Figure 7. The left panel of Figure 7 summarizes the calculated densities alongside the reference experimental data,^52^ as well as the associated relative errors (ϵ_ρ_) calculated as

$$\epsilon_{\rho }=\frac{\rho_{\mathrm{calc}}-\rho_{\exp}}{\rho_{\exp}}$$

**Figure 7:**
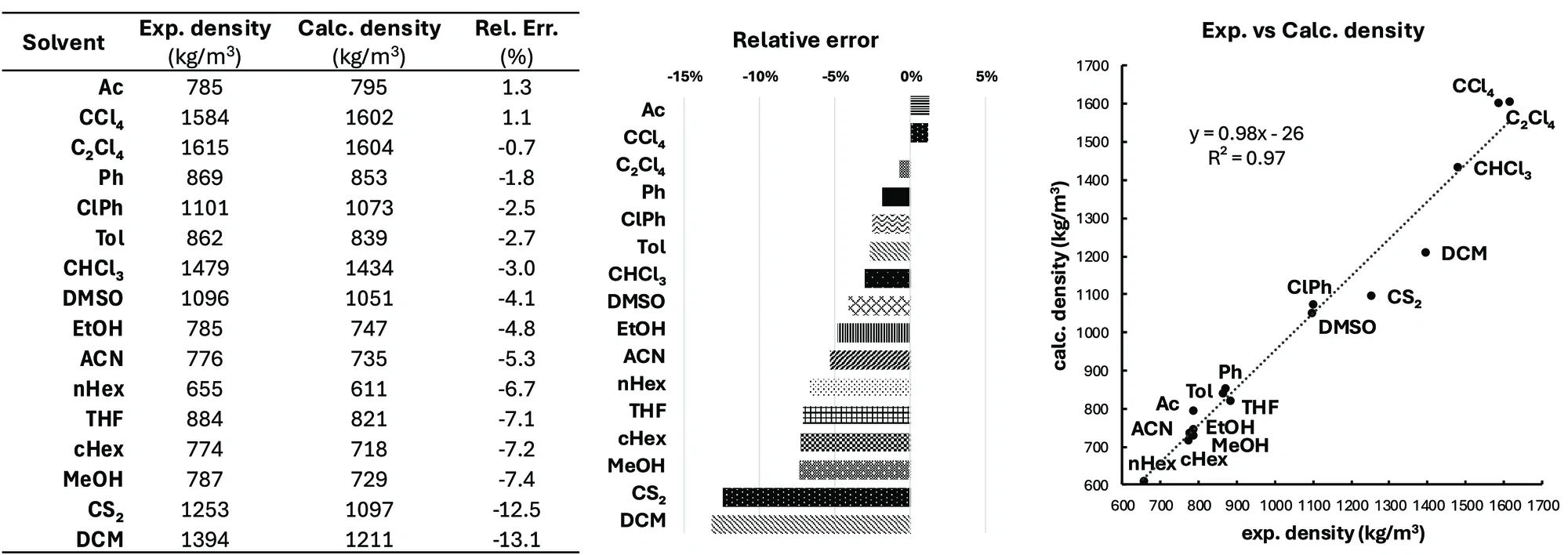
Left panel: table showing experimental and calculated values with relative error. Central panel: bar plot of percentage relative errors, ϵ_ρ_ × 100 (%). Right panel: scatter plot of experimental vs theoretical values, the dashed line represents the linear regression.

The calculated density averages obtained from the 5 ns production run are plotted against the experimental densities in the right panel of Figure 7.

The plot shows a strong correlation between calculated and experimental densities, with relative errors below 10% for almost all solvents. Most data points align closely with the linear regression line. As reported in Figure 7, the regression line approaches the identity line with a coefficient of determination (R^2^) close to 1, indicating good agreement with the experimental data. Notably, for some solvents, the prediction of the density is very good, with errors below 2% (i.e., acetone, tetrachloromethane, tetrachloroethylene, benzene). The slope of the correlation line approaches 1 and the intercept is negligible (26 kg/m^3^), confirming that the force field parameters and simulation conditions are well-suited for predicting densities across diverse molecular liquids.

Interestingly, the absolute relative error in the density prediction correlates with the solvent boiling points (Figure 8). Larger discrepancies occur for low-boiling solvents, likely due to the experimental uncertainty in compressibility data, stronger density fluctuations near the liquid–vapor transition, and the difficulties in simulating near such conditions.

**Figure 8:**
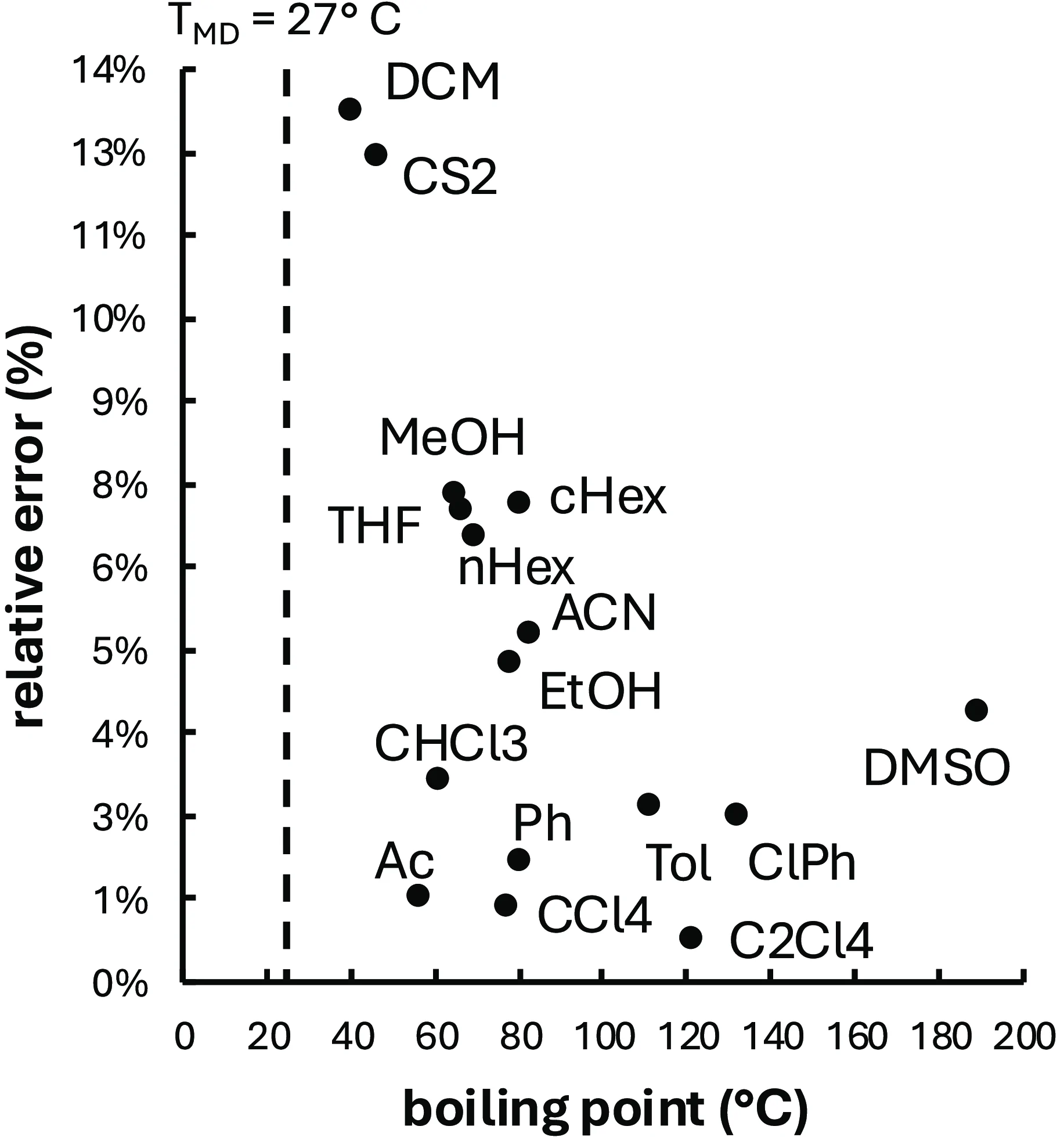
Correlation between the absolute relative error in density prediction and the boiling points of the solvents. Larger errors are observed for low-boiling-point solvents, likely due to increased density fluctuations near the liquid–vapor transition and experimental uncertainties in isothermal compressibility measurements under ambient conditions.

A second source of inaccuracy lies in the parametrization of intermolecular interactions, including van der Waals, hydrogen bonding, π–π stacking, dipole–dipole interactions, and others. Q-Force does not evaluate such parameters; thus, their accuracy practically depends on the selected force field.

Finally, note that if an exact density is required, it is possible to select a frame matching the experimental density and run NVT or NVE simulations from it. No unexpected or unusually high safety hazards were encountered in this tutorial, since the workflow does not involve experimental laboratory operations. All activities are performed in silico using standard scientific software.

## 4 Simulating an UV–vis Absorption Spectrum

Moving on to the simulation of the UV–vis absorption spectrum, we seek to capture solute–solvent interactions, which are essential for a realistic description of spectral features in solution. TD-DFT combined with an implicit solvent model, such as the polarizable continuum model (PCM),^55^ efficiently accounts for the average dielectric response of the solvent and provides good estimates of solvatochromic shifts at low computational cost.^56^ Unfortunately, PCM does not capture specific solute–solvent interactions nor the dynamical fluctuations of the local environment that contribute to spectral broadening. To describe these effects explicitly, an atomistic representation of the solvent is required, which can be obtained from MD trajectory analysis.

Starting from a single indigo molecule embedded in a chloroform solvent box, the system was simulated using GROMACS following the protocol described above (see workflow script in Figure 6). After an initial conjugate-gradient energy minimization, we equilibrated the system for 200 ps in the NVT ensemble and for 200 ps in the NPT ensemble, running afterward a 2 ns NPT production simulation at *T* = 300 K and *P* = 1 bar. All GROMACS commands and input files are provided in the .

With VMD, we extracted 20 indigo microstates at 100 ps intervals, including the first solvation shell. The solvation shell comprises the chloroform molecules nearest to indigo. Its dimension was estimated to be 7 Å based on the VMD-analysis of the indigo-chloroform partial *g*(*r*). A representative solvation shell is shown in Figure 9.

**Figure 9:**
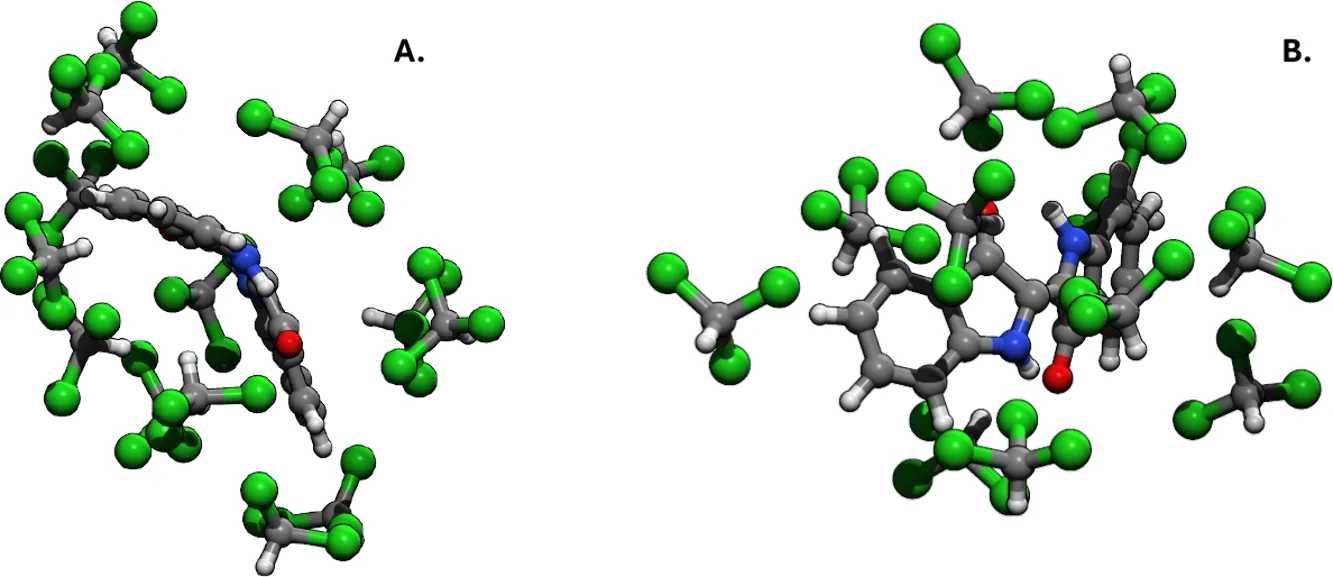
Two frames (A and B) taken from the MD trajectory of indigo in chloroform showing the first solvation shell.

TD-DFT calculations at the B3LYP/6–31G­(d,p) level were performed for each solvated microstate to compute UV–vis spectra. The B3LYP functional, combined with the 6–31G­(d,p) basis set is a widely used, computationally efficient approach for UV–vis spectra of organic molecules. Although hybrid functionals such as B3LYP may not always provide quantitatively accurate excitation energies, they often yield reliable qualitative trends and spectral shapes, which are the primary focus of this tutorial. However, it should be noted that TD-DFT limitations are known for charge-transfer states^57^ and for excited states with double-excitation character.^58^ A detailed discussion of the accuracy and limitations of different functionals can be found in the literature.^59,60^ The individual spectra (see for more details) were algebraically summed with equal weights (i.e., assuming equal statistical relevance of each microstate) to produce the overall spectrum shown in Figure 10, which closely matches the experimental data in peak position and shape. Notably, the shoulder around 560 nm is reproduced, as well as the overall bandwidth of the main absorption peak. Importantly, the agreement with the experimental spectrum emerges only after summing the contributions of all microstates: as shown in Figure 10 (bottom), no individual spectrum accurately captures the absorbance profile of indigo.

**Figure 10:**
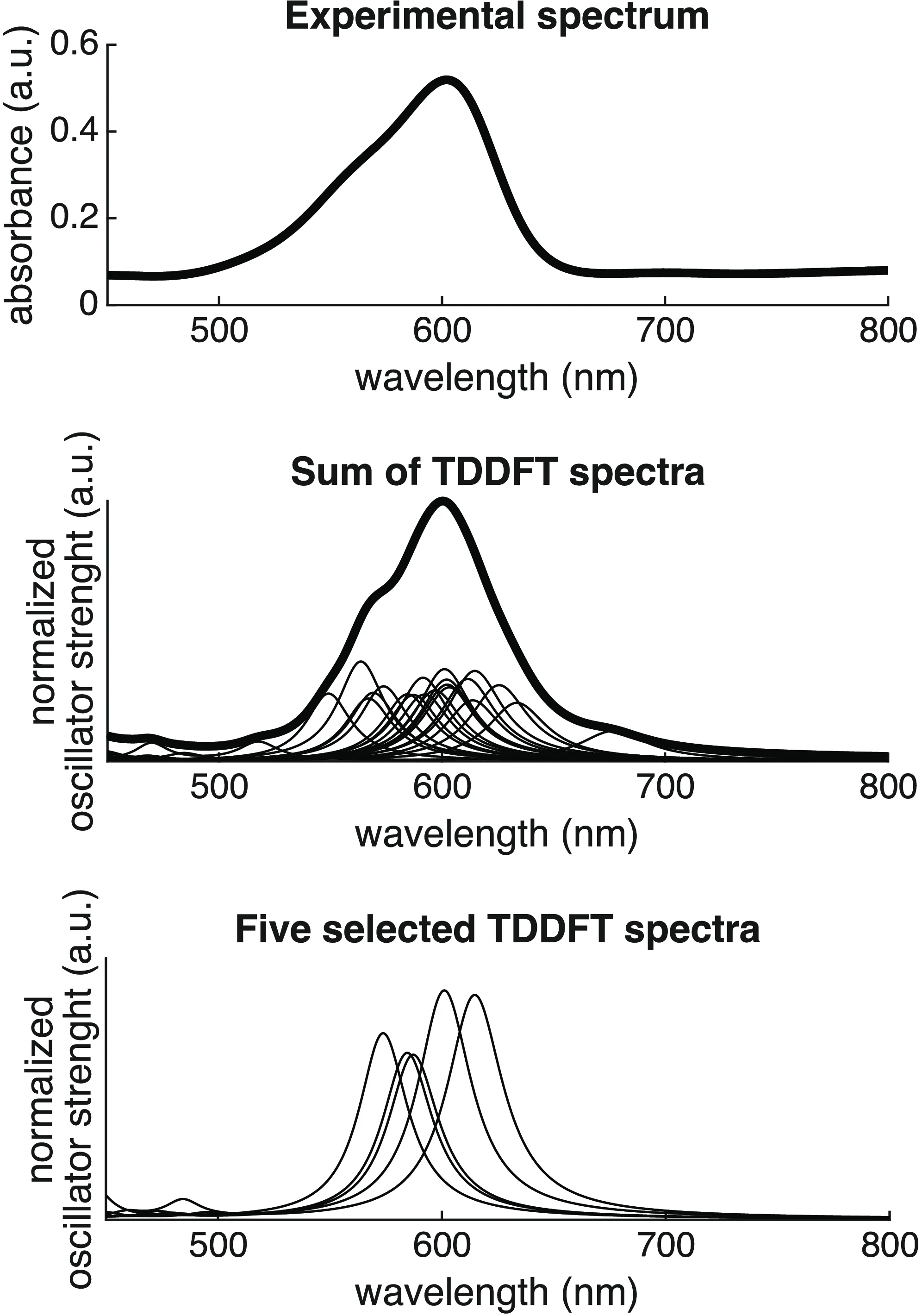
(Top) Experimental UV absorption spectrum of indigo measured at room temperature in chloroform at a concentration of 5 × 10^–5^ M using a 1 cm optical path length. (Middle) Summation of TD-DFT (B3LYP/6–31G­(d,p)) spectra calculated for individual solvated microstates. (Bottom) TD-DFT spectra of the individual microstates. The close agreement between the summed spectrum and the experimental data highlights the accuracy of the combined MM/MD and TD-DFT approach, whereas individual microstates alone show limited agreement with the experimental data.

Finally, Figure 11 provides a schematic overview of the complete workflow, from molecular model construction to UV–vis spectrum generation, and can serve as a practical checklist for student assignments and for setting up future molecular simulations. To support dissemination and promote further research, we have made available all the solvents modeled in this study in a dedicated GitHub repository, along with the necessary simulation boxes and associated files. This repository contains all the components required to incorporate these solvents into GROMACS simulations and can be accessed at https://github.com/cippee/SolventSafari-for-GROMACS.

**Figure 11:**
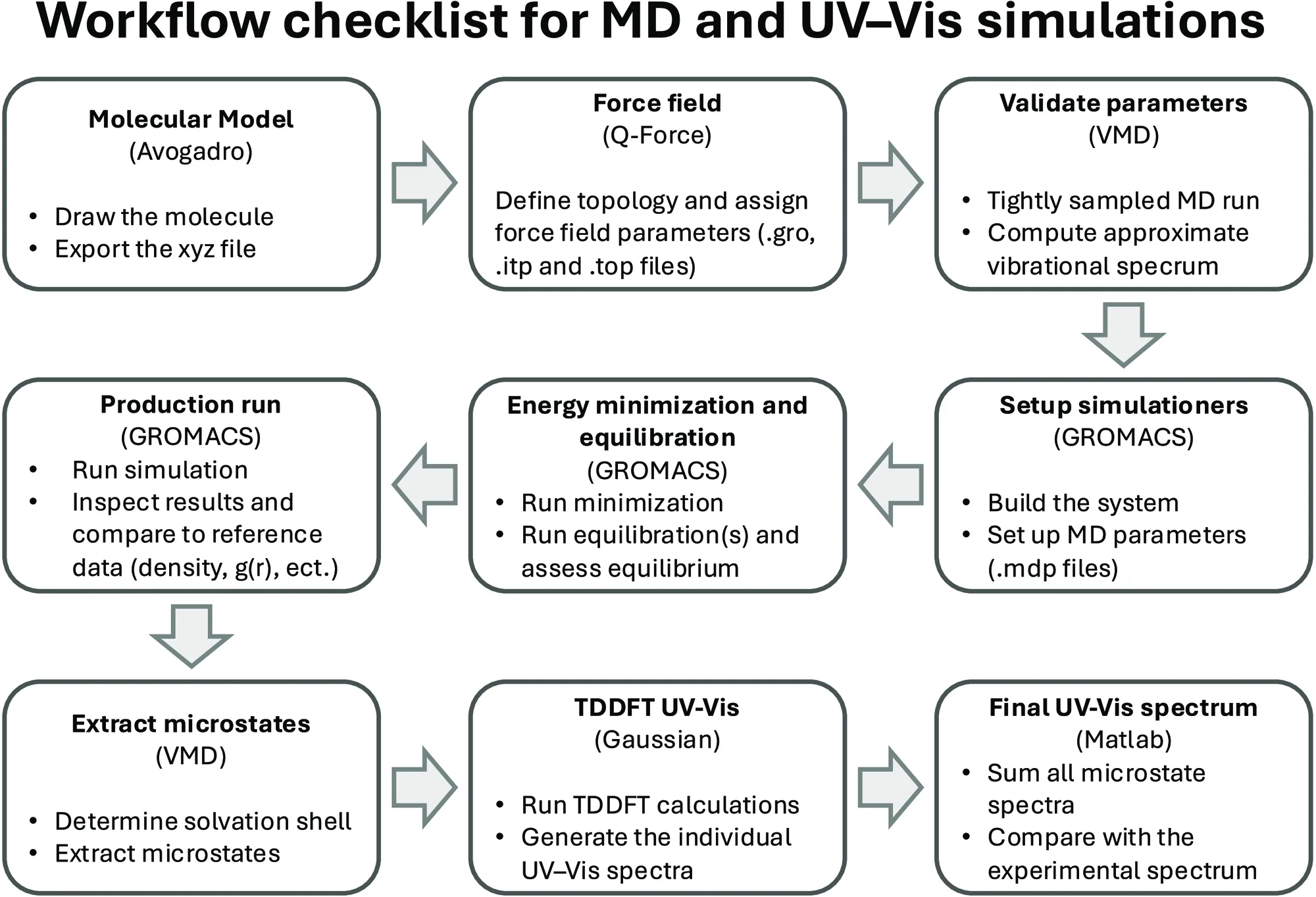
Schematic overview of the complete simulation workflow discussed in this tutorial.

## 5 Conclusions

This tutorial accompanies students through a complete simulation workflow focusing on molecular materials and provides teachers with material ready for laboratory activities. By walking through the construction of topologies, validation of solvent models, and definition of microstates for spectroscopic calculations, this work emphasizes the reasoning behind methodological choices, also providing the necessary software commands.

Through a set of organic solvents, students can understand how force-field parameters translate into measurable properties such as density and structural organization (radial distribution functions). The example of indigo solvated in chloroform further illustrates how MD and TD-DFT can be combined to interpret UV–vis data, demonstrating the role of solvation in spectral shaping.

All the files, examples, and solvent boxes released alongside this tutorial are designed to reduce barriers for classroom adoption. The methodology can be extended to new molecules with reasonable effort. We hope this resource facilitates broader adoption of MD simulations by students in computational and physical chemistry.

## Supplementary Material





## Notes

Archive containing examples of complete GROMACS setups for MD simulations; Details on GitHub repository contents; Details on UV–vis plots and TD-DFT calculations (PDF)
