# Supplementary material for: From GROMACS Molecular Dynamics Simulations to Electronic Absorption Spectra: A Tutorial for Small Molecules in Organic Solvents
Source: J Chem Educ. 2026 Sep 1;103(10):5441–54. doi: 10.1021/acs.jchemed.6c00154 (PMC13679877; doi:10.1021/acs.jchemed.6c00154)
Supplement: Supplementary file 1 [file ed6c00154_si_001.pdf]

Supplementary Information

From GROMACS molecular dynamics simulations to electronic absorption spectra: a tutorial for small molecules in organic solvents

Chiara Picarelli, Giuseppina Raffaini*, Matteo Tommasini*

Dipartimento di Chimica, Materiali e Ingegneria Chimica Giulio Natta, Politecnico di Milano, Piazza Leonardo da Vinci, 32 – 20133 Milano (Italy)

* email: giuseppina.raffaini@polimi.it; matteo.tommasini@polimi.it

Lesson plan for instructors

Molecular simulations are increasingly integrated into graduate-level curricula in physical chemistry, materials science, and computational chemistry. While molecular dynamics (MD) is often introduced through selected classes of molecules (*e.g.*, proteins, carbon nanostructures), students may encounter difficulties when applying these methods to arbitrary organic molecules and solvents, which is a common scenario in fields such as physical chemistry, materials science, and spectroscopy. This tutorial addresses these difficulties by providing a physically motivated and reproducible workflow that connects force-field construction, MD simulations, and spectroscopic observables. The approach emphasizes conceptual understanding over software-specific optimization, supporting the development of transferable skills essential for modern chemical education.

Suitable courses

This tutorial is primarily intended for upper-level undergraduate and graduate courses in physical chemistry, computational chemistry, and materials science, where students are expected to engage with theoretical and computational methods flanking the analysis of experimental data. It may also be adopted in advanced laboratory courses where computational activities complement experimental spectroscopic measurements, or in specialized PhD-level modules on molecular simulations. The modular structure of the tutorial, with self-contained sections on force-field construction (Section 3), MD protocol (Sections 2 and 3), and spectroscopic analysis (Section 4), allows instructors to selectively incorporate individual components according to course objectives and time constraints.

Required prior knowledge

Students would benefit from prior coursework in physical chemistry, including basic thermodynamics, statistical mechanics, and molecular spectroscopy, in particular UV-Vis absorption spectroscopy. Familiarity with classical mechanics and potential energy surfaces is helpful, as is exposure to basic quantum chemistry concepts such as geometry

optimization and vibrational analysis. Practical skills required include basic command-line proficiency (navigating directories and managing files in a Unix-like environment) and general familiarity with molecular structure visualization software. All commands and scripts required to reproduce the MD simulations presented in this tutorial are provided explicitly in the SI and in the accompanying GitHub repository, so that students with limited prior experience with MD software can nonetheless follow the workflow step by step.

Expected pace of instruction

The tutorial is delivered in three modules. The duration required to cover each module (ranging from 2 to 4 hours) could be adjusted based on students' backgrounds and available computational resources.

Session 1. Conceptual foundations and force-field construction: Core concepts of molecular dynamics (timestep, thermodynamic ensembles, nonbonded interactions, and periodic boundary conditions) are introduced, with guided discussion of force-field components and atom types. Students set up their working environment, inspect the provided topology files, and run an energy minimization.

Session 2. Solvent model construction and structural validation: Students follow a step-by-step workflow to construct a solvent box, run NVT and NPT equilibration, and analyze structural properties including density and radial distribution functions, comparing results with reference experimental data.

Session 3. Solute-solvent simulation and spectroscopic observables: The workflow is extended to a solute-solvent system (indigo in chloroform). Students extract solvation-shell microstates from the MD trajectory and interpret ensemble-averaged UV-Vis absorption spectra obtained from TD-DFT calculations. Due to the computational cost of TD-DFT, representative precomputed trajectories and electronic-structure results may be provided for in-class analysis.

Homework assignments may include running TD-DFT calculations on selected configurations, assessing how simulation parameters affect structural and spectroscopic observables, and preparing written or oral reports (see below).

Assessment of student learning

Student learning can be assessed through problem-based assignments in which students are asked to set up, justify, and analyze an MD simulation of a selected small organic molecule in a solvent of their choice. Depending on class proficiency and available computational resources, the instructor may choose whether to provide force-field parameter files.

Conceptual understanding may be evaluated through written reports or oral presentations covering: the theoretical basis of MD, justification of methodological decisions (*e.g.*, choice of timestep, thermostat, and cutoff scheme), and interpretation of simulation results. Practical competence may be assessed by: examining input files; qualitative validation of force-field parameters by comparing DFT-computed IR spectra with VMD-derived MD spectral densities; interpretation of structural observables such as radial distribution

functions and density convergence. Spectroscopic understanding can be assessed by asking students to discuss differences among the experimental UV-Vis absorption spectrum, the individual TD-DFT spectra for selected microstates, and the ensemble-averaged simulated spectrum.

S1. Examples of complete GROMACS setup for MD simulations

This archive contains three directories, each providing the complete set of input files required to run the GROMACS molecular dynamics (MD) mentioned in the main article.

The directories are organized as follows:

- **ethanol_testff**: input files used to generate the vibrational density of states via the velocity autocorrelation function, analyzed using VMD.
- **ethanol_solvent**: input files used to construct and equilibrate a pure solvent box.
- **indigo_chloroform**: input files used to simulate the solvation of one indigo molecule in chloroform and generate trajectories for subsequent spectroscopic analysis.

Each directory includes the following files:

- EtOH.gro: final equilibrated structure
- EtOH.top: system topology
- EtOH.itp: force field "included topology" file containing atom types and parameters
- *.mdp: molecular dynamics parameter files for energy minimization, NVT and NPT equilibration, and NPT production
- workflow.sh: bash script automating the simulation pipeline (minimization, NVT equilibration, NPT equilibration, and NPT production run)

These examples are intended as practical reference for reproducing or adapting the MD simulation protocol discussed in this tutorial.

S2. Information on the solvents

The archive available at github.com/cippee/SolventSafari-for-GROMACS provides all GROMACS force field parameter files (*itp*) and pre-equilibrated solvent box structure files (*gro*) ready for use. In addition, the molecular dynamics parameter files (*mdp*) required to reproduce these solvent boxes are included.

Solvent-specific information is summarized in plain text data sheet (Fig. S2). For each solvent, the following information is reported:

- system name, common name, and chemical formula
- literature values density and isothermal compressibility, with appropriate references
- number of molecules (N) and simulation box edge length (l)
- force field generation method, including the level of theory used for the parametrization

- complete simulation protocol, including energy minimization, equilibration NVT and NPT, and production runs
- average thermodynamic properties (potential and kinetic energy, volume, temperature, pressure, and density) computed using the ‘gmx energy’ tool, along with the corresponding GROMACS error estimates

```
********************************************************************************
January 2026

C. Picarelli - chiara.picarelli@polimi.it

Department of Chemistry, Materials and Chemical Engineering Giulio Natta
(Building 6)

Politecnico di Milano
Piazza Leonardo Da Vinci 32
20133 Milano (Italy)

********************************************************************************

Ethanol - EtOH - CH3CH2OH

d = 0.785 g/cm3 [1]
B = 1.153e-4 bar-1 [1]
N = 1000
l = 4.6 nm

Force field topology was parametrized with Q-Force [2] at PBEPBE/6-31+G(D) level of theory.
MD simulations were performed with GROMACS 2022.4 [3].

Simulation details:
Energy minimization: conjugate gradient algorithm.
Equilibration I: NVT ensemble, 500 ps, 1 fs, 300 K, V-rescale thermostat.
Equilibration II: NPT ensemble, 500 ps, 1 fs, 300 K, 1 bar, V-rescale thermostat and C-rescale barostat.
MD production run: NPT ensemble, 5 ns, 1 fs, 300 K, 1 bar, V-rescale thermostat and C-rescale barostat.

Thermodynamic observables, including volume, temperature, pressure, and density, were computed from the
production trajectory using the gmx energy tool.

Quantity                   Average   Err.Est.       RMSD  Tot-Drift
-------------------------------------------------------------------------------
Potential                  37853.5         49    354.046   -263.588  (kJ/mol)
Kinetic En.                33687.6        7.2    280.443    3.97474  (kJ/mol)
Total Energy               71541.1         47    436.561   -259.614  (kJ/mol)
Temperature                300.158      0.064    2.49876  0.0354157  (K)
Pressure                   12.4288        4.5    561.954    32.2694  (bar)
Volume                     102.436       0.19   0.772883  -0.954337  (nm^3)
Density                    746.836        1.4    5.61519    6.91372  (kg/m^3)



[1] - Y. Marcus "The Properties of Solvents", Wiley 1998.
[2] - Sami, S.; Menger, M. F. S. J.; Faraji, S.; Broer, R.; Havenith, R. W. A., Q-Force: Quantum Mechanic
Augmented Molecular Force Fields. Journal of Chemical Theory and Computation 2021, 17 (8), 4946-4960.
[3] - https://doi.org/10.5281/zenodo.7323393.
```

Fig. S1 - Example content of a solvent specification sheet (EtOH_specs.txt) included in the specs.zip archive. Each file reports bibliographic data, simulation parameters, and the average thermodynamic properties (volume, temperature, pressure, and density) computed from the NPT production trajectory. All files follow the same structure for consistency and ease of comparison.

The reported data refer to the equilibrated regime of the 5 ns NPT production simulations performed with GROMACS 2022.4. References to the relevant literature and software tools are included at the end of each file.

In addition, plots of potential and kinetic energy, volume, temperature, pressure, and density over the equilibration and production stages are provided.

S3. Simulation of UV-Vis spectra

The simulated UV-Vis spectra S(E) of each solvated microstate can be obtained by summing normalized Lorentzian functions L(E, E_k, Γ), centered at the TDDFT-computed transition energies (E_k), with line broadening parameter (Γ), weighted by the TDDFT-computed oscillator strength (f_k):

$$S(E) = \sum_k f_k \cdot L(E,E_k,\Gamma) = \sum_k f_k \cdot \frac{\Gamma}{2\pi} \frac{1}{(E - E_k)^2 + \Gamma^2/4}$$

Individual S(E) curves can be obtained by using, *e.g.*, Excel spreadsheets or Matlab/Octave scripts. The transformation from energy units (E, typically in eV) to wavelength (λ, typically in nm) can be done for plotting purposes (Fig. 10), considering that λ(nm) ≈ 1240 / E(eV).

An example of the Gaussian input file employed for the TD-DFT calculations used to reproduce the UV–Vis spectrum shown in Fig. 10 is reported below. The excited-state calculations were performed at the B3LYP/6-31G(d,p) level of theory, requesting 30 singlet excited states.

```
%NProc=20
%Mem=31740mb
%chk=ind_chcl3_frame_100.chk
#P b3lyp/6-31G(d,p) td(Nstates=30)

ind_chcl3_frame_100

0 1
  C   19.362798690795898   19.362798690795898   19.362798690795898
  C   18.003144264221188   19.616575241088867   19.597879409790039
  C   16.914828300476071   18.680902481079102   19.319417953491211
  C   17.395088195800781   17.526082992553711   18.708709716796875
  C   18.699085235595703   17.252889633178711   18.493818283081055
  C   19.709941864013668   18.190656661987308   18.757240295410156
  H   20.005485534667965   20.110548019409183   19.722211837768555
  H   17.636666297912598   20.424791336059570   20.193582534790039
  H   15.929775238037108   18.863435745239258   19.649154663085938
  H   16.653398513793942   16.716703414916992   18.505424499511722
  N   20.957141876220700   17.676092147827148   18.419832229614258
  C   19.373249053955075   16.003526687622067   18.023706436157227
  C   21.922620773315426   15.494264602661136   18.067554473876953
  N   21.776699066162113   14.156427383422853   17.701772689819336
  C   23.057321548461914   15.504392623901367   19.121372222900387
```

C	23.530206680297852	14.127996444702150	19.180536270141605
O	23.311491012573242	16.457876205444336	19.895959854125977
C	22.736335754394531	13.356576919555666	18.249574661254883
C	24.383541107177734	13.527727127075192	20.094730377197266
C	24.683326721191403	12.167308807373047	19.895298004150394
C	20.835527420043942	16.345550537109375	18.060132980346680
O	18.901256561279293	14.941900253295898	17.708658218383789
C	23.969915390014645	11.412403106689453	18.927736282348633
C	22.963022232055661	11.937950134277344	18.129978179931641
H	22.372446060180664	11.312767028808592	17.437505722045902
H	25.404546737670891	11.733715057373049	20.623809814453129
H	24.262348175048825	10.315315246582033	18.842550277709964
H	25.156480789184574	14.073204040527344	20.692743301391605
H	21.852350234985344	17.970056533813473	18.742044448852539
H	20.853845596313473	13.881750106811525	17.416450500488281
C	20.475431442260739	15.612119674682615	21.986564636230472
H	21.020032882690430	16.340501785278320	21.287210464477539
Cl	21.400171279907230	15.578184127807621	23.487730026245114
Cl	20.312845230102536	14.021814346313473	21.246660232543949
Cl	18.903821945190430	16.361886978149414	22.176065444946289
C	20.723592758178707	14.568864822387694	13.913726806640627
H	20.679981231689450	14.868467330932615	12.826261520385746
Cl	22.237825393676754	13.598596572875973	14.078695297241213
Cl	19.468103408813477	13.423799514770506	14.449762344360353
Cl	20.570123672485348	16.103368759155273	14.856287002563478
C	16.499724388122555	23.439085006713867	18.817037582397464
H	16.180908203125000	22.455774307250977	19.156173706054688
Cl	15.922083854675291	24.666875839233398	20.051916122436527
Cl	16.121777534484860	23.884412765502933	17.136997222900394
Cl	18.284835815429684	23.149679183959961	19.060369491577148
C	26.409639358520508	12.479093551635742	16.585105895996097
H	25.559827804565430	11.980543136596678	17.124959945678711
Cl	27.164335250854489	10.886135101318358	16.007938385009766
Cl	27.534858703613278	13.364809036254885	17.735391616821289
Cl	25.910953521728516	13.433488845825195	15.236211776733398
C	13.861186981201170	21.403615951538086	22.117919921875000

H	12.823109626770016	21.274450302124023	22.387918472290039
Cl	14.392502784729000	23.008731842041012	22.549575805664062
Cl	14.041113853454588	21.076810836791992	20.383274078369144
Cl	14.572860717773434	20.125589370727539	22.993946075439453
C	22.597152709960938	21.001255035400394	22.393285751342773
H	22.538398742675781	21.064043045043945	23.519119262695312
Cl	22.546819686889645	19.226535797119141	22.052820205688477
Cl	24.108846664428711	21.752252578735352	22.025285720825195
Cl	21.265151977539062	21.865777969360348	21.437587738037113
C	26.487112045288086	7.869360923767091	20.704763412475589
H	27.421096801757805	7.346343994140625	20.802156448364261
Cl	25.921947479248040	7.656391143798829	18.874467849731442
Cl	26.627689361572266	9.540834426879883	21.200027465820312
Cl	25.512660980224609	6.826615333557129	21.750717163085941
C	21.393354415893551	20.488481521606445	14.949930191040041
H	22.208299636840820	19.937778472900387	15.421026229858398
Cl	21.048177719116211	19.726560592651367	13.359510421752931
Cl	21.846635818481445	22.169553756713867	14.666830062866213
Cl	19.938680648803707	20.395261764526367	16.031263351440433
C	19.305023193359371	19.197439193725589	24.628835678100586
H	19.971830368041989	18.494276046752930	24.136650085449222
Cl	20.322790145874020	20.577413558959964	25.427116394042969
Cl	18.497039794921875	18.303073883056637	25.990278244018558
Cl	18.295087814331055	19.830743789672852	23.365463256835938
C	18.062663078308102	10.241220474243164	16.874826431274414
H	17.646821022033688	10.600574493408201	15.906162261962892
Cl	16.765647888183590	10.085289001464846	18.170827865600586
Cl	19.304473876953125	11.378381729125978	17.238121032714844
Cl	18.752901077270508	8.607000350952148	16.542266845703121
C	16.320519447326657	19.212078094482418	15.117839813232424
H	17.053018569946286	18.527236938476562	15.519966125488281
Cl	15.782266616821286	18.462095260620117	13.671077728271486
Cl	17.095909118652340	20.834474563598633	14.696365356445316
Cl	14.982188224792479	19.325464248657223	16.288194656372070
C	26.982187271118157	17.089826583862308	19.827611923217777
H	25.970493316650391	16.659643173217773	19.564113616943359

Cl	27.831724166870121	17.703485488891602	18.346174240112305
Cl	27.831895828247067	15.563051223754885	20.352890014648438
Cl	26.774475097656250	18.345026016235352	21.011705398559570
C	15.394588470458981	14.903598785400392	21.752243041992184
H	14.590287208557127	14.268739700317383	21.877891540527347
Cl	14.743779182434080	16.410137176513672	21.134611129760742
Cl	16.604556083679199	14.134094238281250	20.685447692871097
Cl	16.159389495849606	15.217088699340822	23.375381469726566
C	15.996216773986815	13.614572525024412	16.671365737915039
H	16.842761993408203	13.843935012817383	17.335092544555668
Cl	15.336169242858887	15.176355361938475	16.327936172485352
Cl	14.901356697082518	12.703834533691408	17.848846435546875
Cl	16.174534797668453	12.612867355346680	15.223011016845705
C	26.291261672973626	17.616243362426758	15.555730819702148
H	27.066541671752926	16.878137588500977	15.818113327026369
Cl	27.061897277832028	19.095298767089844	15.237604141235352
Cl	24.937526702880856	17.773572921752930	16.829929351806641
Cl	25.637527465820309	16.875892639160156	14.083456039428713
