# Supplementary material for: From GROMACS Molecular Dynamics Simulations to Electronic Absorption Spectra: A Tutorial for Small Molecules in Organic Solvents
Source: J Chem Educ. 2026 Sep 1;103(10):5441–54. doi: 10.1021/acs.jchemed.6c00154 (PMC13679877; doi:10.1021/acs.jchemed.6c00154)
Supplement: Supplementary file 2 [file ed6c00154_si_002.docx]

**Supplementary Information**

## **From GROMACS molecular dynamics simulations to electronic absorption spectra: a tutorial for small molecules in organic solvents**

## Chiara Picarelli, Giuseppina Raffaini*, Matteo Tommasini*

## Dipartimento di Chimica, Materiali e Ingegneria Chimica Giulio Natta, Politecnico di Milano, Piazza Leonardo da Vinci, 32 – 20133 Milano (Italy)

* email: [giuseppina.raffaini@polimi.it](mailto:giuseppina.raffaini@polimi.it); [matteo.tommasini@polimi.it](mailto:matteo.tommasini@polimi.it)

**Lesson plan for instructors**

Molecular simulations are increasingly integrated into graduate-level curricula in physical chemistry, materials science, and computational chemistry. While molecular dynamics (MD) is often introduced through selected classes of molecules (*e.g.*, proteins, carbon nanostructures), students may encounter difficulties when applying these methods to arbitrary organic molecules and solvents, which is a common scenario in fields such as physical chemistry, materials science, and spectroscopy. This tutorial addresses these difficulties by providing a physically motivated and reproducible workflow that connects force-field construction, MD simulations, and spectroscopic observables. The approach emphasizes conceptual understanding over software-specific optimization, supporting the development of transferable skills essential for modern chemical education.

**Suitable courses**

This tutorial is primarily intended for upper-level undergraduate and graduate courses in physical chemistry, computational chemistry, and materials science, where students are expected to engage with theoretical and computational methods flanking the analysis of experimental data. It may also be adopted in advanced laboratory courses where computational activities complement experimental spectroscopic measurements, or in specialized PhD-level modules on molecular simulations. The modular structure of the tutorial, with self-contained sections on force-field construction (Section 3), MD protocol (Sections 2 and 3), and spectroscopic analysis (Section 4), allows instructors to selectively incorporate individual components according to course objectives and time constraints.

**Required prior knowledge**

Students would benefit from prior coursework in physical chemistry, including basic thermodynamics, statistical mechanics, and molecular spectroscopy, in particular UV-Vis absorption spectroscopy. Familiarity with classical mechanics and potential energy surfaces is helpful, as is exposure to basic quantum chemistry concepts such as geometry optimization and vibrational analysis. Practical skills required include basic command-line proficiency (navigating directories and managing files in a Unix-like environment) and general familiarity with molecular structure visualization software. All commands and scripts required to reproduce the MD simulations presented in this tutorial are provided explicitly in the SI and in the accompanying GitHub repository, so that students with limited prior experience with MD software can nonetheless follow the workflow step by step.

**Expected pace of instruction**

The tutorial is delivered in three modules. The duration required to cover each module (ranging from 2 to 4 hours) could be adjusted based on students' backgrounds and available computational resources.

*Session 1. Conceptual foundations and force-field construction*: Core concepts of molecular dynamics (timestep, thermodynamic ensembles, nonbonded interactions, and periodic boundary conditions) are introduced, with guided discussion of force-field components and atom types. Students set up their working environment, inspect the provided topology files, and run an energy minimization.

*Session 2. Solvent model construction and structural validation*: Students follow a step-by-step workflow to construct a solvent box, run NVT and NPT equilibration, and analyze structural properties including density and radial distribution functions, comparing results with reference experimental data.

*Session 3. Solute-solvent simulation and spectroscopic observables*: The workflow is extended to a solute-solvent system (indigo in chloroform). Students extract solvation-shell microstates from the MD trajectory and interpret ensemble-averaged UV-Vis absorption spectra obtained from TD-DFT calculations. Due to the computational cost of TD-DFT, representative precomputed trajectories and electronic-structure results may be provided for in-class analysis.

Homework assignments may include running TD-DFT calculations on selected configurations, assessing how simulation parameters affect structural and spectroscopic observables, and preparing written or oral reports (see below).

**Assessment of student learning**

Student learning can be assessed through problem-based assignments in which students are asked to set up, justify, and analyze an MD simulation of a selected small organic molecule in a solvent of their choice. Depending on class proficiency and available computational resources, the instructor may choose whether to provide force-field parameter files.

Conceptual understanding may be evaluated through written reports or oral presentations covering: the theoretical basis of MD, justification of methodological decisions (*e.g.*, choice of timestep, thermostat, and cutoff scheme), and interpretation of simulation results. Practical competence may be assessed by: examining input files; qualitative validation of force-field parameters by comparing DFT-computed IR spectra with VMD-derived MD spectral densities; interpretation of structural observables such as radial distribution functions and density convergence. Spectroscopic understanding can be assessed by asking students to discuss differences among the experimental UV-Vis absorption spectrum, the individual TD-DFT spectra for selected microstates, and the ensemble-averaged simulated spectrum.

**S1. Examples of complete GROMACS setup for MD simulations**

This archive contains three directories, each providing the complete set of input files required to run the GROMACS molecular dynamics (MD) mentioned in the main article.

The directories are organized as follows:

- **ethanol_testff**: input files used to generate the vibrational density of states via the velocity autocorrelation function, analyzed using VMD.
- **ethanol_solvent**: input files used to construct and equilibrate a pure solvent box.
- **indigo_chloroform**: input files used to simulate the solvation of one indigo molecule in chloroform and generate trajectories for subsequent spectroscopic analysis.

Each directory includes the following files:

- EtOH.gro: final equilibrated structure
- EtOH.top: system topology
- EtOH.itp: force field “included topology” file containing atom types and parameters
- *.mdp: molecular dynamics parameter files for energy minimization, NVT and NPT equilibration, and NPT production
- workflow.sh: bash script automating the simulation pipeline (minimization, NVT equilibration, NPT equilibration, and NPT production run)

These examples are intended as practical reference for reproducing or adapting the MD simulation protocol discussed in this tutorial.

**S2. Information on the solvents**

The archive available at [github.com/cippee/SolventSafari-for-GROMACS](https://github.com/cippee/SolventSafari-for-GROMACS) provides all GROMACS force field parameter files (*itp*) and pre-equilibrated solvent box structure files (*gro*) ready for use. In addition, the molecular dynamics parameter files (*mdp*) required to reproduce these solvent boxes are included.

Solvent-specific information is summarized in plain text data sheet (Fig. S2). For each solvent, the following information is reported:

- system name, common name, and chemical formula
- literature values density and isothermal compressibility, with appropriate references
- number of molecules (N) and simulation box edge length (l)
- force field generation method, including the level of theory used for the parametrization
- complete simulation protocol, including energy minimization, equilibration NVT and NPT, and production runs
- average thermodynamic properties (potential and kinetic energy, volume, temperature, pressure, and density) computed using the ‘gmx energy’ tool, along with the corresponding GROMACS error estimates

Fig. S1 - Example content of a solvent specification sheet (EtOH_specs.txt) included in the specs.zip archive. Each file reports bibliographic data, simulation parameters, and the average thermodynamic properties (volume, temperature, pressure, and density) computed from the NPT production trajectory. All files follow the same structure for consistency and ease of comparison.

The reported data refer to the equilibrated regime of the 5 ns NPT production simulations performed with GROMACS 2022.4. References to the relevant literature and software tools are included at the end of each file.

In addition, plots of potential and kinetic energy, volume, temperature, pressure, and density over the equilibration and production stages are provided.

**S3. Simulation of UV-Vis spectra**

The simulated UV-Vis spectra S(E) of each solvated microstate can be obtained by summing normalized Lorentzian functions L(E, E_k_, Γ), centered at the TDDFT-computed transition energies (E_k_), with line broadening parameter (Γ), weighted by the TDDFT-computed oscillator strength (f_k_):

$$S\left( E \right)=\sum_{k} f_{k}\cdot L(E,E_{k},\Gamma)=\sum_{k} f_{k}\cdot\frac{\Gamma}{2\pi}\frac{1}{\left( E-E_{k} \right)^{2}+\Gamma^{2}/4}$$

Individual S(E) curves can be obtained by using, *e.g.*, Excel spreadsheets or Matlab/Octave scripts. The transformation from energy units (E, typically in eV) to wavelength (λ, typically in nm) can be done for plotting purposes (Fig. 10), considering that λ(nm) ≈ 1240 / E(eV).

An example of the Gaussian input file employed for the TD-DFT calculations used to reproduce the UV–Vis spectrum shown in Fig. 10 is reported below. The excited-state calculations were performed at the B3LYP/6-31G(d,p) level of theory, requesting 30 singlet excited states.

```
%NProc=20
%Mem=31740mb
%chk=ind_chcl3_frame_100.chk
#P b3lyp/6-31G(d,p) td(Nstates=30)

ind_chcl3_frame_100

0 1
   C   19.362798690795898   19.362798690795898   19.362798690795898
   C   18.003144264221188   19.616575241088867   19.597879409790039
   C   16.914828300476071   18.680902481079102   19.319417953491211
   C   17.395088195800781   17.526082992553711   18.708709716796875
   C   18.699085235595703   17.252889633178711   18.493818283081055
   C   19.709941864013668   18.190656661987308   18.757240295410156
   H   20.005485534667965   20.110548019409183   19.722211837768555
   H   17.636666297912598   20.424791336059570   20.193582534790039
   H   15.929775238037108   18.863435745239258   19.649154663085938
   H   16.653398513793942   16.716703414916992   18.505424499511722
   N   20.957141876220700   17.676092147827148   18.419832229614258
   C   19.373249053955075   16.003526687622067   18.023706436157227
   C   21.922620773315426   15.494264602661136   18.067554473876953
   N   21.776699066162113   14.156427383422853   17.701772689819336
   C   23.057321548461914   15.504392623901367   19.121372222900387
   C   23.530206680297852   14.127996444702150   19.180536270141605
   O   23.311491012573242   16.457876205444336   19.895959854125977
   C   22.736335754394531   13.356576919555666   18.249574661254883
   C   24.383541107177734   13.527727127075192   20.094730377197266
   C   24.683326721191403   12.167308807373047   19.895298004150394
   C   20.835527420043942   16.345550537109375   18.060132980346680
   O   18.901256561279293   14.941900253295898   17.708658218383789
   C   23.969915390014645   11.412403106689453   18.927736282348633
   C   22.963022232055661   11.937950134277344   18.129978179931641
   H   22.372446060180664   11.312767028808592   17.437505722045902
   H   25.404546737670891   11.733715057373049   20.623809814453129
   H   24.262348175048825   10.315315246582033   18.842550277709964
   H   25.156480789184574   14.073204040527344   20.692743301391605
   H   21.852350234985344   17.970056533813473   18.742044448852539
   H   20.853845596313473   13.881750106811525   17.416450500488281
   C   20.475431442260739   15.612119674682615   21.986564636230472
   H   21.020032882690430   16.340501785278320   21.287210464477539
  Cl   21.400171279907230   15.578184127807621   23.487730026245114
  Cl   20.312845230102536   14.021814346313473   21.246660232543949
  Cl   18.903821945190430   16.361886978149414   22.176065444946289
   C   20.723592758178707   14.568864822387694   13.913726806640627
   H   20.679981231689450   14.868467330932615   12.826261520385746
  Cl   22.237825393676754   13.598596572875973   14.078695297241213
  Cl   19.468103408813477   13.423799514770506   14.449762344360353
  Cl   20.570123672485348   16.103368759155273   14.856287002563478
   C   16.499724388122555   23.439085006713867   18.817037582397464
   H   16.180908203125000   22.455774307250977   19.156173706054688
  Cl   15.922083854675291   24.666875839233398   20.051916122436527
  Cl   16.121777534484860   23.884412765502933   17.136997222900394
  Cl   18.284835815429684   23.149679183959961   19.060369491577148
   C   26.409639358520508   12.479093551635742   16.585105895996097
   H   25.559827804565430   11.980543136596678   17.124959945678711
  Cl   27.164335250854489   10.886135101318358   16.007938385009766
  Cl   27.534858703613278   13.364809036254885   17.735391616821289
  Cl   25.910953521728516   13.433488845825195   15.236211776733398
   C   13.861186981201170   21.403615951538086   22.117919921875000
   H   12.823109626770016   21.274450302124023   22.387918472290039
  Cl   14.392502784729000   23.008731842041012   22.549575805664062
  Cl   14.041113853454588   21.076810836791992   20.383274078369144
  Cl   14.572860717773434   20.125589370727539   22.993946075439453
   C   22.597152709960938   21.001255035400394   22.393285751342773
   H   22.538398742675781   21.064043045043945   23.519119262695312
  Cl   22.546819686889645   19.226535797119141   22.052820205688477
  Cl   24.108846664428711   21.752252578735352   22.025285720825195
  Cl   21.265151977539062   21.865777969360348   21.437587738037113
   C   26.487112045288086    7.869360923767091   20.704763412475589
   H   27.421096801757805    7.346343994140625   20.802156448364261
  Cl   25.921947479248040    7.656391143798829   18.874467849731442
  Cl   26.627689361572266    9.540834426879883   21.200027465820312
  Cl   25.512660980224609    6.826615333557129   21.750717163085941
   C   21.393354415893551   20.488481521606445   14.949930191040041
   H   22.208299636840820   19.937778472900387   15.421026229858398
  Cl   21.048177719116211   19.726560592651367   13.359510421752931
  Cl   21.846635818481445   22.169553756713867   14.666830062866213
  Cl   19.938680648803707   20.395261764526367   16.031263351440433
   C   19.305023193359371   19.197439193725589   24.628835678100586
   H   19.971830368041989   18.494276046752930   24.136650085449222
  Cl   20.322790145874020   20.577413558959964   25.427116394042969
  Cl   18.497039794921875   18.303073883056637   25.990278244018558
  Cl   18.295087814331055   19.830743789672852   23.365463256835938
   C   18.062663078308102   10.241220474243164   16.874826431274414
   H   17.646821022033688   10.600574493408201   15.906162261962892
  Cl   16.765647888183590   10.085289001464846   18.170827865600586
  Cl   19.304473876953125   11.378381729125978   17.238121032714844
  Cl   18.752901077270508    8.607000350952148   16.542266845703121
   C   16.320519447326657   19.212078094482418   15.117839813232424
   H   17.053018569946286   18.527236938476562   15.519966125488281
  Cl   15.782266616821286   18.462095260620117   13.671077728271486
  Cl   17.095909118652340   20.834474563598633   14.696365356445316
  Cl   14.982188224792479   19.325464248657223   16.288194656372070
   C   26.982187271118157   17.089826583862308   19.827611923217777
   H   25.970493316650391   16.659643173217773   19.564113616943359
  Cl   27.831724166870121   17.703485488891602   18.346174240112305
  Cl   27.831895828247067   15.563051223754885   20.352890014648438
  Cl   26.774475097656250   18.345026016235352   21.011705398559570
   C   15.394588470458981   14.903598785400392   21.752243041992184
   H   14.590287208557127   14.268739700317383   21.877891540527347
  Cl   14.743779182434080   16.410137176513672   21.134611129760742
  Cl   16.604556083679199   14.134094238281250   20.685447692871097
  Cl   16.159389495849606   15.217088699340822   23.375381469726566
   C   15.996216773986815   13.614572525024412   16.671365737915039
   H   16.842761993408203   13.843935012817383   17.335092544555668
  Cl   15.336169242858887   15.176355361938475   16.327936172485352
  Cl   14.901356697082518   12.703834533691408   17.848846435546875
  Cl   16.174534797668453   12.612867355346680   15.223011016845705
   C   26.291261672973626   17.616243362426758   15.555730819702148
   H   27.066541671752926   16.878137588500977   15.818113327026369
  Cl   27.061897277832028   19.095298767089844   15.237604141235352
  Cl   24.937526702880856   17.773572921752930   16.829929351806641
  Cl   25.637527465820309   16.875892639160156   14.083456039428713
```
